# Endovascular image-guided sampling of tumor-draining veins provides an enriched source of oncological biomarkers

**DOI:** 10.3389/fonc.2023.916196

**Published:** 2023-03-17

**Authors:** Anobel Tamrazi, Srividya Sundaresan, Aishwarya Gulati, Frederick J. Tan, Vibhor Wadhwa, Bjarne R. Bartlett, Luis A. Jr. Diaz

**Affiliations:** ^1^ Division of Vascular and Interventional Radiology, Palo Alto Medical Foundation, Redwood City, CA, United States; ^2^ Department of Clinical Research, Dignity Health, Sequoia Hospital, Redwood City, CA, United States; ^3^ Department of Radiology, Thomas Jefferson University Hospital, Philadelphia, PA, United States; ^4^ Department of Embryology, Carnegie Institution, Baltimore, MD, United States; ^5^ Division of Interventional Radiology, NewYork-Presbyterian/Weill Cornell Medical Center, New York, NY, United States; ^6^ Department of Molecular Biosciences and Bioengineering, University of Hawaíi at Mānoa, Honolulu, HI, United States; ^7^ Department of Medicine, Memorial Sloan Kettering Cancer Center, New York, NY, United States

**Keywords:** tumor-draining vein, tumor-proximal, oncological biomarker, circulating tumor cell (CTC), microRNA (miRNA), circulating tumor DNA (ctDNA), minimally invasive, liquid biopsy

## Abstract

**Introduction:**

Circulating tumor-derived biomarkers can potentially impact cancer management throughout the continuum of care. This small exploratory study aimed to assess the relative levels of such biomarkers in the tumor-draining vascular beds in patients with solid tumors compared to levels in their peripheral veins.

**Methods:**

Using an endovascular image-guided approach, we obtained blood samples from peripheral veins and other vascular compartments–including the most proximal venous drainage from solid tumors–from a set of nine oncology patients with various primary and metastatic malignancies. We then interrogated these samples for a panel of oncological biomarkers, including circulating tumor cells (CTCs), exosome-derived microRNAs (miRNAs), circulating tumor DNA (ctDNA) mutations, and certain cancer-related proteins/biochemical markers.

**Results:**

We found substantially higher levels of CTCs, certain miRNAs, and specific ctDNA mutations in samples from vascular beds closer to the tumor compared with those from peripheral veins and also noted that some of these signals were altered by treatment procedures.

**Discussion:**

Our results indicate that tumor-proximal venous samples are highly enriched for some oncological biomarkers and may allow for more robust molecular analysis than peripheral vein samples.

## Introduction

Tumor cells release various factors into the circulation that can be collected *via* the peripheral blood, including extracellular vesicles (EVs) or exosomes containing microRNA (miRNA), circulating tumor cells (CTCs), proteins, and circulating tumor DNA (ctDNA) (1–3). This ‘liquid biopsy’ can characterize a tumor in a manner comparable to a tissue biopsy and holds significant promise for orchestrating approaches for early detection, diagnosis, assessment of minimal residual disease, genotyping tumors, and tracking therapeutic response and resistance (4–8). Several actionable mutations in genes with diagnostic and prognostic significance, such as *EGFR* for lung cancer, and *KRAS* and *BRAF* for colorectal cancer have been well-studied from ctDNA extracted from peripheral blood samples (9–12). However, many limitations hold liquid biopsy back from its full potential, and tissue biopsy remains the standard of care in current clinical oncology practice (2, 3, 7, 8). One issue is that liquid biopsy efforts have largely focused on peripheral venous blood samples. In general, these contain very low concentrations of tumor-derived biomarkers that are difficult to detect in standard assay platforms. As a result, highly sensitive methods have been required to detect such low levels, with most of these efforts currently placed on ctDNA. Furthermore, methods to detect these rare molecules can introduce errors and result in false positive findings (2, 8). If the yield obtained while sampling could possibly be amplified for a variety of these biomarkers, it would result in significant progress in the applications of liquid biopsies.

Several surgical and endoscopic studies report higher levels of certain oncological biomarkers in the tumor-draining veins of solid malignancies when compared to peripheral circulation (13–20). This implies that the anatomy of the vascular system might dictate the relative concentrations of various biomarkers in the different vascular beds. There are also reports that the properties and distributions of these markers in vascular beds proximal to a tumor may provide information on mutational status, metastatic potential, and immune evasion mechanisms (15, 17, 19, 21, 22). Additionally, as Buscail et al. noted in their review (23), certain biomarkers such as tumor-proximal CTCs may be more relevant as diagnostic and prognostic markers in certain types of cancers than in others. However, the biology of tumor-associated biomarkers is not fully characterized, since most of these reports from surgical literature are limited by a single point of venous sampling during tumor resection (24, 25).

To address the challenges and the gaps in knowledge described above, we tested a hypothesis that analyzing blood from the most proximal venous drainage of a tumor–using standard minimally-invasive image-guided endovascular techniques with low profile soft catheters–would provide the highest yield of oncological biomarkers. Blood sampling from tumor-draining vasculature is not a new concept and is well-documented in the medical literature. It has been used for several decades to localize functional non-malignant tumors such as pituitary adenomas, parathyroid adenomas, adrenal adenomas, and insulinomas (26–29), but has yet to be fully explored with emerging molecular biomarkers in malignant tumors.

In this study, we compared the relative yields and features of key biomarkers from tumor-draining or tumor-proximal veins, peripheral samples, and other vascular beds in several patients with known malignancies at different stages of disease and treatment to better understand the suitability of selective and targeted blood sampling for oncological applications. We present our data through a case series report.

## Methods

### Patients

Nine patients with known malignancies of various types (detailed below) were identified and sampled at different stages in their course of treatment during endovascular procedures required for their care after informed consent under an IRB-approved protocol. Please refer to **Table 1** for a summary.

1) Patient 1 was a 52-year-old male with Cowden Syndrome, diagnosed with an unresectable pancreatic neuroendocrine tumor (PNET, T4N1; Ki-67 low proliferative rate of <2%) and a 5.0 x 3.5 cm Stage 1B right renal cell carcinoma (RCC) (**Supplementary Figure 1**).
2) Patient 2 was a 69-yr-old female diagnosed with Stage IV endometrial carcinoma with squamous differentiation (85% endometroid, 15% clear cell) and multiple lung nodules also suspicious for metastasis.
3) Patient 3 was a 34-yr-old male with large infiltrative hepatocellular carcinoma nearly completely replacing right hepatic lobe, with likely metastasis within the lung and associated portal vein thrombosis and ascites, who underwent palliative trans-arterial chemembolization (TACE) (**Supplementary Figure 2**).
4) Patient 4 was a 63-yr-old male with synchronous urothelial carcinoma of the left ureter and bladder, that was locally advanced and high grade.
5) Patient 5 was a 70-year-old female with stage IV high grade serous ovarian carcinoma with extensive metastasis in the left side of the neck and chest, adenopathy including left supraclavicular lymph nodes, left brain parietal cortex, and left upper abdominal wall soft tissues.
6) Patient 6 was a 60-year-old male with a gastrointestinal stromal tumor (GIST) at the lesser curvature of the stomach with extensive metastasis to the liver. This patient underwent a TACE procedure targeting right lobe of the liver tumor burden.
7) Patient 7 was a 58-year-old male with sigmoid adenocarcinoma stage IV with hepatic metastasis, pulmonary nodules, and retroperitoneal metastatic lymphadenopathy (LAD).
8) Patient 8 was a 61-year-old male with a moderate to poorly differentiated pancreatic adenosquamous carcinoma at the tail of the pancreas with metastasis to the liver.
9) Patient 9 was a 51-year-old male with acinar pancreatic carcinoma with metastasis to the liver and soft tissue metastases to both thighs.

**Table 1:** Summary of the clinical details, vascular beds sampled, and biomarkers analyzed in this case series.

Number	Characteristics	Diagnosis/Procedure	Vasculature sampled	Biomarker data given
1	51-yr-old male	Cowden Syndrome, Metachronous Pancreatic Neuroendocrine Tumor, right renal cell carcinoma	PE, PV (TDV1 for PNET) RRV (TPV1 for RCC), LRV, SV	CTCs, miRNA, ctDNA, serotonin, chromogranin
2	69-yr-old female	Endometrial carcinoma, lung metastases	PE, LIIV(TPV2), SIVC (TPV4)	CTCs
3	34-yr-old male	Hepatocellular carcinoma, lung metastases	PE, 1^st^ order small ARHV branch (TDV1), inferior main branch of ARHV (TPV1 of segment 6/7 mass), main branch of MHV (TPV1 of segment 5/8 mass), RCFA, RAHA, RPHA, AA	CTCs
4	63-yr-old male	Synchronous left renal pelvis and left bladder urothelial carcinoma	PE, LRV superior branch (TDV1), LIIV and RIIV (TPV1), RRV (similar to PE)	CTCs
5	70-yr-old female	Ovarian carcinoma, left supraclavicular mass, left abdominal wall soft tissue mass	PE, SVC (TPV4 of neck mass), IIVC (TPV4 of abdominal mass), LCFV (similar to PE)	miRNA, ctDNA
6	60-yr-old male	Gastrointestinal stromal tumor, liver metastases	PE (LCFV considered peripheral vein), RHV (TPV1 of right hepatic lobe mass)	miRNA
7	58-yr-old male	Sigmoid adenocarcinoma, liver and lung metastases, retroperitoneal metastatic lymphadenopathy	PE, MHV1 (TPV1 of segment 8 lesion), MHV2 (similar to PE), RHV (similar to PE)	ctDNA
8	60-yr-old male	Pancreatic adenosquamous carcinoma, liver metastasis	PE, ARHV anatomical variant (TPV2 for segment 6 mass), RHV (similar to PE)	ctDNA
9	51-yr-old male	Pancreatic acinar carcinoma, liver and thigh (soft tissue) metastases	PE, RHV (TPV1)	Lipase: pre-and post-chemoembolization
Control Patient 1	53-yr-old male	Cirrhosis, hernias, ascites - TIPS	PE, RIJ, MHV, RRV, LRV	miRNA
Control Patient 2	47-yr-old male	Factor V Leiden with DVTs – kissing stents and angioplasty	PE, RIJ, RHV, PV	miRNA

Sampled vasculature: AA, aortic arch; ARHV, accessory right hepatic vein; IIVC, infrarenal inferior vena cava; LCFV, left common femoral vein; LIIV, left internal iliac vein; LRV, left renal vein; MHV, middle hepatic vein; PE, peripheral vein; PV, portal vein; RAt, right atrium; RAHA, right anterior hepatic artery; RCFA, right common femoral artery; RHV, right hepatic vein; RIIV, right internal iliac vein; RIJ, right internal jugular; RPHA, right posterior hepatic artery; RRV, right renal vein; SIVC, suprarenal inferior vena cava; TDV, tumor-draining vein; TPV, tumor-proximal vein. Other abbreviations: TIPS, transjugular intrahepatic portosystemic shunt. DVT, deep vein thrombosis.

#### Controls

Control Patient 1 was a 53-year-old male with cirrhosis who presented with multiple hernias (umbilical, periumbilical, inguinal) and uncontrolled ascites. This patient underwent a transjugular intrahepatic portosystemic shunt (TIPS) procedure at our clinic.

Control Patient 2 was a 47-year-old male with a medical history of Factor V Leiden thrombophilia complicated by multiple deep vein thromboses and pulmonary embolisms. This patient underwent a “kissing balloon” angioplasty of the bilateral iliac veins and IVC, placement of kissing stents within the bilateral iliac veins and IVC, and balloon angioplasty of the newly placed stents.

All patients were consented for prospective blood samples to be obtained during their procedures for research purposes as part of an IRB-approved protocol. The sampled vascular beds and biomarkers analyzed in these patients are summarized in **Table 1**. We had four patient samples for CTC (1, 2, 3, and 4), three for EV-derived miRNA (1, 5, 6), four for ctDNA (1, 5, 6, 8), and two for protein biomarker (1, 9) analyses, respectively. As controls for this study, we collected blood from two patients (Control Patient 1 and Control Patient 2) without known malignancies who underwent non-oncology-related endovascular procedures. These control samples were used for the microRNA analyses only (signals evaluated were not tumor-specific) since CTCs and ctDNA were not expected to be present in individuals without a cancer diagnosis. The FDA-approved CellSearch^®^ platform (https://www.cellsearchctc.com/) was used for detecting CTCs in cancer patients. The ctDNA for blood and tissue somatic mutational analyses were performed by Personal Genome Diagnostics (PGDx) as per their standard protocols. All patients were consented for prospective blood samples to be obtained during their procedures for research purposes as part of an IRB-approved protocol.

### Sampling from tumor-draining and tumor-proximal veins

Close vascular access to tumor-draining and tumor-proximal beds was achieved through a standard endovascular image-guided method with soft catheters that is safe and minimally invasive. The venous system was entered through cannulation of the common femoral vein, internal jugular vein, or portal vein with a low profile (< 2 mm diameter) soft tip catheter for sampling. The arterial system was entered similarly through standard endovascular techniques of the common femoral artery. The positioning of the catheter was confirmed with radiographic imaging and contrast injection. Blood samples (up to 10 ml) from various vascular beds were obtained using this approach.

### Sample processing and biomarker analyses

#### CTC analysis

For CTC analysis, the CellSearch^®^ system that targets CD45-, EpCAM+, Cytokeratin 8, 18+ and/or 19+ cells was employed. Whole blood samples were collected in CellSave Preservative tubes - Streck™ BCT vacutainer tubes and kept at room temperature until processed, usually within 96 hours. The CellSearch^®^ assay used ferrofluid nanoparticles coated with antibodies to magnetically separate cells expressing the specific surface markers. Separated cells with +DAPI staining and CD45- were then presented as possible CTCs.

#### Exosome and microRNA isolation and analysis

Blood samples were collected in K_2_EDTA containing tubes, kept at 4°C, and processed within 2 hours. MicroRNAs were assessed from plasma and tumor tissue. Assays for a panel of microRNAs (miR-16-5p, miR-21-5p, miR-34a-5p, miR-122-5p, miR-126, miR-145-5p, miR-146a, miR-150-5p, miR-155-5p, miR-223-3p, and miR-375, henceforth referred to without the ‘p’ or ‘arm’ designation) were used for analysis across multiple fractions of samples from each location. Blood was processed to plasma within a maximum of 120 minutes of collection by centrifugation at 800xg for 15 minutes (2500S). The supernatant was then diluted at least 1:1 with filtered Phosphate Buffered Saline (PBS) and successively centrifuged at 2500xg for 15 minutes (2500P), 10,000xg for 30 minutes (10kP), and 110,000xg for 70 minutes (110kP). Pellets from each step were retained for analysis, as was supernatant from the final centrifugation. With established ultracentrifugation techniques, it is expected that there will be a decreasing presence of larger EVs and apoptotic bodies in the 110kP relative to the 2500P fraction. Pellets from each centrifugation step were resuspended in 1xPBS by pipetting up and down 10 times, vortexing for thirty seconds, incubating on ice for 20 minutes, and repeating the pipetting and vortexing steps. RNA was isolated from each fraction using the RNA isolation kit - Biofluids from Exiqon, Woburn, MA (Catalog #300112, #300113)); spiked-in control RNA (synthetic cel-miR-39-3p) and glycogen were used as reference and carrier, respectively.

For miRNA analysis of tissue samples, samples were placed in RNA-specific containers without formaldehyde on dry ice. RNA was extracted from biopsied tissue using QiaZol reagent (Qiagen, Catalog #79306) and tissue disruption with Lysing Matrix D (MP Biomedicals/Fisher Scientific, Catalog # MP116933050), followed by on-column RNA cleanup with mirVana miRNA extraction reagents (Thermo Fisher Scientific, Catalog #AM1560). Equal volumes of extracted RNA were placed into stem-loop reverse transcription/quantitative PCR assays with the following TaqMan^®^ assay ID primers and probes from Thermo Fisher Scientific: miR-16 (000391), miR-21 (000397), miR-34a (000426), miR-122 (002245), miR-126 (000451), miR-145 (002278), mi-146a (000468), miR-150 (000473), miR-155 (002623), miR-223 (002295), miR-375 (000564). Quantitation threshold (Cq) values were volume corrected and normalized to the spiked-in control or endogenously expressed miR-16-5p or snRNA U6 (30). As this is a relative quantitation method, microRNA levels in the TDVs/TPVs are expressed as fold change over peripheral values. For Patients 1 and 8, transmission electron microscopy (TEM) was performed on each fraction from the plasma preparation with uranyl acetate staining in the Johns Hopkins Neurology facility after dialyzing away the phosphate-containing buffer.

#### ctDNA analyses

For Patients 5, 7, and 8, blood samples were collected in K_2_EDTA containing tubes and sent to Personal Genome Diagnostics (PGDx) (Baltimore, MD) on ice. Plasma was extracted by PGDx for their next generation sequencing (NGS)-based analysis using their PLASMA*SELECT*-R^™^ panel of 63 well-characterized cancer genes according to their established protocols. These data are represented as mutant fractional abundance in the tested samples, derived from the number of distinct mutant reads in the total number of distinct reads for each gene. For Patient 1, we first performed targeted sequencing on the patient’s pancreatic neuroendocrine tumor (PNET) (Foundation Medicine, Cambridge, MA) and whole exome sequencing of the renal cell carcinoma (RCC) tissue samples for somatic variants (PGDx, CANCER*Xome* ^™^ Analysis) to define potential targets in our ctDNA assay. The targeted sequencing of the PNET tissue did not reveal any somatic mutations. But the whole-exome sequencing of the RCC picked up several somatic mutations out of which the bonafide and clinically relevant ones are mentioned in **Supplementary Table 1**. These somatic mutations were used as targets for a digital PCR-based assay performed at Dr. Luis Diaz’s lab at Johns Hopkins University. For this, blood samples were collected in K_2_EDTA containing tubes, kept at 4°C, and processed within 2 hours. Circulating cell-free DNA was extracted from samples using the QIAamp Circulating Nucleic Acid Kit (Qiagen, Catalog #55114). Qubit dsDNA HS Assay Kit (Life Technologies, Catalog #Q32854) was used to determine the concentration of purified DNA. Extracted DNA was analyzed with droplet digital PrimePCR™ assay (BioRad, Catalog #100-31246 and #100-31249). The mutants were calculated as a percentage of the total amount of DNA.

#### Proteomic/biochemical marker analyses

For Patient 1, whole blood samples were placed into EDTA containing tubes (serotonin) and plain vacutainer tubes without anticoagulant (chromogranin A) and analyzed for the levels of these proteins according to standard methods by the Johns Hopkins Hospital laboratory and Quest Diagnostics Nichols Institute. Lipase levels for Patient 9 were measured according to standard methods by the Johns Hopkins Hospital laboratory.

## Results

### Nomenclature and hypothesis

**Figure 1:**
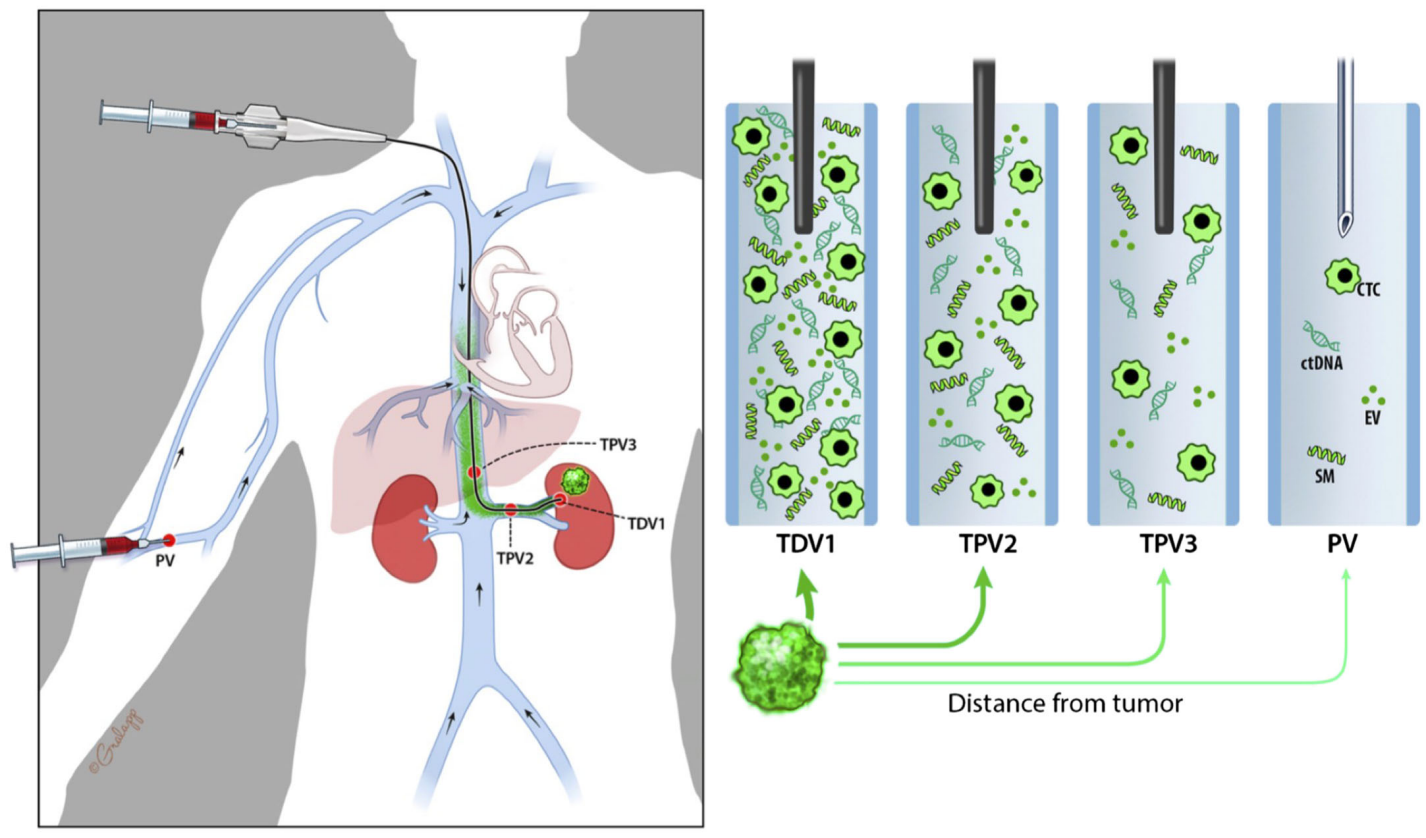
Schematic diagram representing nomenclature of tumor-draining and tumor-proximal veins and dilution in biomarker signal (in green) when sampled away from the tumor (green mass in the left kidney). Whole-body view is in the left panel and signal dilution at different sampling stations is depicted in the right panels. We refer to the most proximal branch of a vein draining the tumor as the tumor-draining vein (TDV1), and then in ascending order as tumor-proximal vein (TPV) 2, 3, etc., the further away it is from the tumor. We hypothesize that biomarker signals would be the highest in the TDV1, followed by TPV2, then TPV3, and so on.

To clearly indicate the proximity of the sampled vascular beds to the tumor, we propose the following nomenclature: the primary branch of the vein that drains the solid tumor is designated the 1^st^ order tumor-draining vein (referred to as TDV1). The vascular beds sampled downstream from this TDV1 are referred to as the tumor-proximal veins (TPVs) of the 2^nd^ (TPV2), 3^rd^ (TPV3), or 4^th^ (TPV4) order (Schematic in **Figure 1**; whole body view in left panel and sampling stations in right panels). The ascending numerical order indicates the further the TPV is from the tumor. Our hypothesis is that the TDV1 will have the highest concentration of biomarkers, followed by TPV2, TPV3, and so on (**Figure 1**, right panels), due to the dilution expected when the signal-enriched blood from the TDV1 mixes with other unenriched venous blood at the site of sampling, downstream of the solid tumor (**Figure 1**, left panel). We tested this hypothesis in the case series presented below.

### Case series and biomarkers

The current case series consists of nine oncology and two control patients, with a highlight on Patient 1, a 52-year-old male with Cowden Syndrome, an unresectable pancreatic neuroendocrine tumor (PNET) and a right renal cell carcinoma (RCC, Stage IB) (images of both tumors in **Supplementary Figure 1**). Patient 1’s samples were analyzed for all of the biomarkers in our study: liquid biopsy focused on CTCs, ctDNAs, exosome-derived microRNAs, and proteomic analyses, as well as renal mass tissue for microRNA, and separately, whole-exome sequencing. Other patients’ samples were assayed for one or more of these biomarkers, as indicated in **Table 1**. We present the data based on the type of biomarkers (CTC, ctDNA, etc.) analyzed from various vascular beds in our patient cohort. It is important to note that this was a proof-of-concept study to evaluate behavior and concentration differences of various tumor-related signals, rather than a clinical trial to detect sensitivity of a particular platform or focused on one particular biomarker. It was not possible to obtain replicate blood samples from a single patient at each vascular location due to the limits of blood obtained per patient in our IRB. Hence, we examined data from several patients per biomarker (CTC, microRNA, and ctDNA) to get a better sense of the enrichment levels in different vascular beds in individuals with known malignancies. Additionally, for each patient, their own peripheral blood samples served as internal controls for the biomarker being tested and were compared with blood samples obtained closer to their solid tumor at the same time. Any promising results from this pilot study need to be evaluated in a future larger study with a bigger patient cohort and requisite controls for statistical analyses.

### CTCs (Patients 1, 2, 3, and 4)

For all patients, whole blood was collected from various vascular venous and arterial compartments (**Table 1**) using an image-guided approach we refer to as “targeted liquid biopsy,” and CTCs enumerated as described in the materials and methods section.

#### Patient 1

(i) For PNET, portal vein (PV)=TDV1 and (ii) for RCC, right renal vein (RRV)=TPV1.

**Figure 2:**
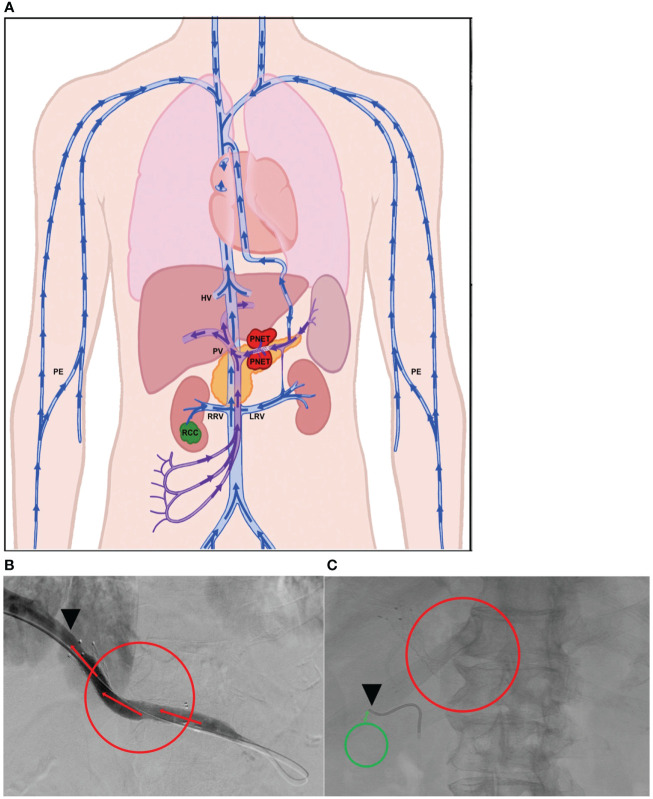
Tumor locations and vascular venous compartments for Patient 1. Schematic of pancreatic neuroendocrine tumor (PNET) and renal cell carcinoma (RCC) tumor locations and various vascular venous compartments sampled with targeted liquid biopsy **(A)**. Intra-procedural image demonstrating improved venous drainage through the portal vein (PV, TDV1 for the PNET) post-stenting, which was initially collapsed by extrinsic compression by the large PNET **(B)**. Intra-procedural image demonstrating venous sampling with a soft endovascular catheter of the right renal vein (RRV, TPV1 for the RCC) **(C)**. Red mass and circles correspond to the PNET, green mass and circles correspond to the RCC, black arrowheads show location of venous sampling from the TDV/TPV of the two tumors. Additional vascular beds sampled are labeled as hepatic vein (HV), peripheral vein (PE), and left renal vein (LRV). TDV – tumor-draining vein, TPV – tumor-proximal vein. Arrows show direction of venous drainage within the vascular venous beds.

**Figure 3:**
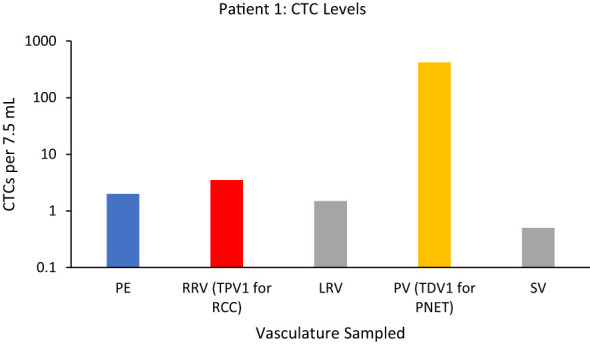
CTC levels in the tumor-draining/proximal and peripheral veins of Patient 1. The mean (n=2) value of circulating tumor cells (CTCs) detected in the portal vein, TDV1 of the PNET, was 419 CTCs per 7.5 ml compared with < 4 CTCs per 7.5 ml detected in any other vascular compartment sampled: right renal vein (RRV), left renal vein (LRV), splenic vein (SV), peripheral vein (PE). Please note that while RRV is the TPV1 for the patient’s other tumor, the renal cell carcinoma (RCC), these tumor cells were not efficiently detected by the CellSearch assay; therefore, the numbers reported for this sample are likely an undercount. The y-axis is on a log scale.

Panel (A) in **Figure 2** is a schematic of the venous drainage anatomy of these two tumors. Panels (B) and (C) and show images of catheter access for both tumors. Since the CellSearch^®^ platform for CTC extraction/enumeration is dependent on epithelial and cytokeratin markers, as expected, only CTCs from the patient’s PNET (and not from the RCC) were detected and enumerated (Maertens 2017). There was a 210-fold higher concentration of CTCs in the TDV1 of the PNET relative to all other vascular compartments sampled, including a peripheral sample (PE) from the right arm (**Figure 3**).

#### Patient 2

Left uterine vein (not sampled)=TDV1, left internal iliac vein (LIIV)=TPV2, left common iliac vein (not sampled)=TPV3, suprarenal inferior vena cava (IVC)=TPV4.

**Figure 4:**
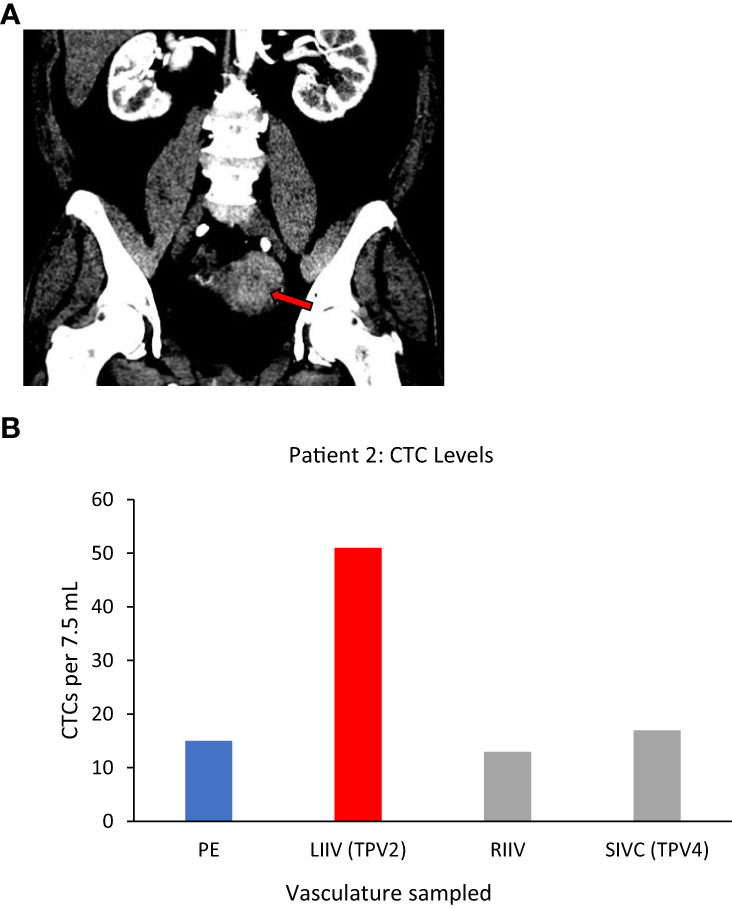
Tumor locations and CTC levels for Patient 2. Panel **(A)** shows enhancing biopsy-proven endometrial cancer mass within the left side of the uterus (red arrow). Panel **(B)** shows CTC levels from some of the vascular beds sampled, including the left internal iliac vein (LIIV), the TPV2 for this mass. Highest levels were in TPV2, with a possible dilutional effect the further the sampled vascular bed was from the tumor. CTC yield shown is mean (n=2) from 7.5 ml of sampled blood. TPV2 is in red, other sampled veins, the suprarenal inferior vena cava (SIVC, TPV4) the right internal iliac vein (RIIV) and are in grey, and the peripheral sample (PE) is in blue.

Patient 2, who had a large left-sided uterine carcinoma (**Figure 4A**), showed a less dramatic enrichment compared to Patient 1, but still had 3-fold higher CTC levels in the left internal iliac vein (LIIV; TPV2) over the peripheral vein from the right arm (**Table 1** for diagnosis and vasculature sampled, **Figure 4B** for CTC levels). The TDV1 here would be the left uterine vein; therefore, a TPV sample obtained just downstream of the TDV1 would be expected to have some signal dilution. Consistent with this hypothesis (**Figure 1**), we noted that the enrichment in the CTC levels in the TPV2, compared to peripheral samples, dropped drastically as we sampled further downstream from the TDV1 of the uterine mass. This was apparent even in the TPV4 sample, suggesting that the diminishing gradient in CTC levels as we sampled a vein further downstream from the TDV1–without the CTC-enriched blood filtering through the liver, spleen, or lungs–was most likely a dilutional, rather than a filtration or degradation effect.

#### Patient 3

Small branch of the accessory right hepatic vein (ARHV) close to the tumor=TDV1, main branch of the middle hepatic vein (MHV)=TPV1 of the segment 5/8 mass, main (inferior) branch of the ARHV=TPV1of segment 6/7 mass.

**Figure 5:**
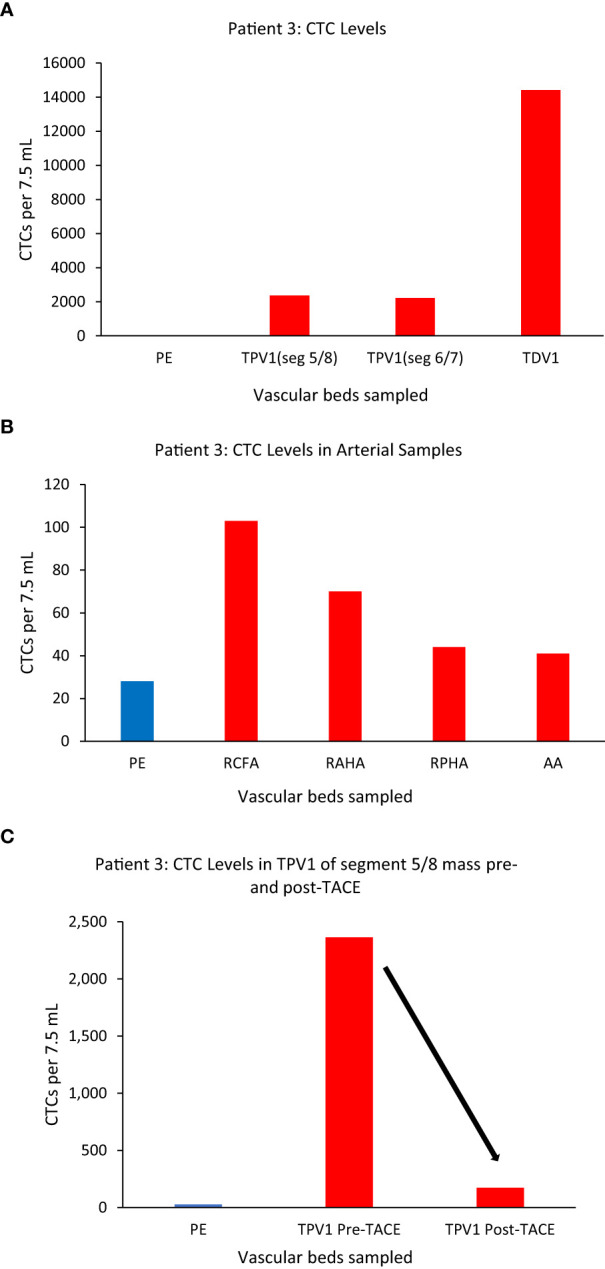
CTC levels in the tumor-draining and peripheral veins of patient 3. A small branch of the accessory right hepatic vein (ARHV) close to the tumor was considered as TDV1 for the segment 5/8 mass, the middle hepatic vein (MHV) as TPV1 of the segment 5/8 mass, and an inferior branch of the ARHV as the TPV1of segment 6/7 mass. Panel **(A)** shows CTC levels were: 2364 in the TPV1 of the segment 5/8 mass, 2213 in the TPV1 of the segment 6/7 mass, and 14412 in the TDV1 (TDV and TPVs in red) compared to peripheral sample (PE, in blue). Panel **(B)** shows CTC values in various arterial vascular beds with substantially less CTC enrichment in contrast to venous sampling downstream of liver tumors *via* the hepatic veins as noted in **(A)**. Right common femoral artery (RCFA), right anterior hepatic artery (RAHA), right posterior hepatic artery (RPHA) and the aortic arch (AA) are in red and peripheral vein sample (PE) is in blue. Panel **(C)** shows a reduction in CTC levels in the TPV1 of the segment 5/8 mass after trans-arterial chemoembolization (TACE) of this mass from 2364 (pre-TACE) to 173 (post-TACE). All CTC values are per 7.5 ml of blood.

We again noticed a substantial enrichment of CTCs in the TDVs and TPVs of Patient 3, who was diagnosed with hepatocellular carcinoma (HCC). This patient had large HCC tumors throughout the right hepatic lobe including in segment 5/8 (drained by the middle hepatic vein (MHV), and in segment 6/7 (drained by the right hepatic vein (RHV)) (**Supplementary Figures 2A, B**). Venous samples were collected from multiple compartments and analyzed for CTC levels (**Table 1** for vasculature sampled). Samples from MHV and an inferior accessory branch of the RHV (ARHV) which are the TPV1s of the segment 5/8 and 6/7 masses (catheter venous access in **Supplementary Figures 2C, D**), respectively, both demonstrated around 80-fold higher CTC levels compared with peripheral blood from the right arm (**Figure 5A**). In venous samples obtained closer to the tumor, i.e., from the 1^st^ order ARHV branch which would be the TDV1 (**Supplementary Figure 3A**), an even greater enrichment of around 500-fold in CTC levels (**Figure 5A**) was noted. On the other hand, arterial samples collected from different regions had much lower CTC counts compared to the TDVs and TPVs and were not substantially enriched compared with peripheral levels (**Figure 5B**).

Additionally, we sampled the same TPV1 of the segment 5/8 tumor of Patient 3 on the same day, before and after a trans-arterial chemoembolization (TACE) (50 mg doxorubicin, 10 mg mitomycin and lipiodol embolization oil) deposited selectively in segments 5/8 *via* the right anterior hepatic artery branch (**Supplementary Figures 3B, C**). This time, the CTC numbers in the same location of the TPV1 (draining the tumor-containing segment of liver that was just chemoembolized) dropped drastically from 2364 to 173 immediately post-TACE (**Figure 5C**). As expected, the levels in the RHV (draining the untreated segment 6/7 tumor) did not decline after TACE of segment 5/8 (data not shown), consistent with CTC levels being unaffected in segment samples where the TACE cocktail was not deposited. In this case, we were able to: a) sample arterial and venous blood from multiple vascular beds and show the highest CTC enrichment in the TDV1 (~500-fold), and b) sample at multiple time points before and after a treatment procedure, tracking successful chemoembolization treatment targeting efficacy.

#### Patient 4

Superior branch of the left renal vein (LRV)=TDV1, left and right internal iliac veins=TPV1s, right renal vein=similar to peripheral vein (PE).

**Figure 6:**
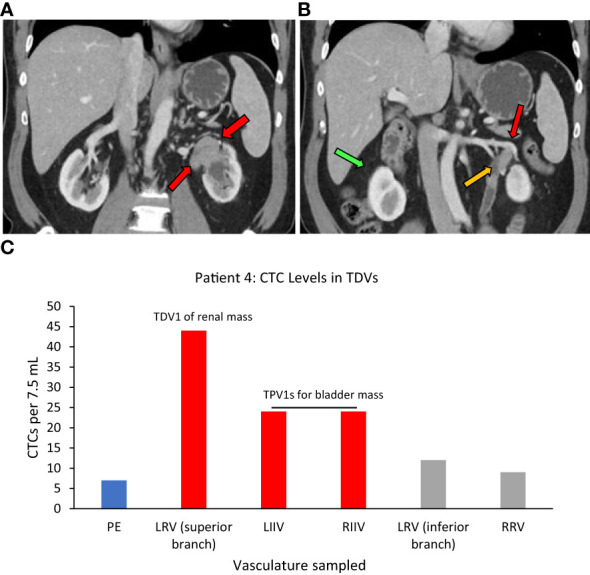
Tumor locations and CTC levels for Patient 4. Panel **(A)** shows tissue biopsy-proven synchronous renal pelvic urothelial carcinoma on the left side (red arrows point to the tumor). Red and yellow arrows in **(B)** point to the superior branch of the left renal vein (LRV) (designated TDV1) and inferior branch of the LRV (not tumor-draining, similar to peripheral vein (PE)) for the renal mass, respectively, and the green arrow points to the right renal vein (RRV). Panel **(C)** shows CTC levels in the tumor-draining and peripheral veins of this patient with the highest levels seen in TDV1 for the renal mass and the left and right internal iliac veins (LIIV and RIIV) which are the TPV1s for the bladder mass. PE – peripheral vein. TDV1s are in red, TPVs in grey, and PE in blue.

One more example of CTC enrichment in the tumor-draining vasculature was in the case of Patient 4, who had synchronous left urothelial renal and bladder carcinoma (verified by tissue biopsy) (**Figure 6A**). Here, of the two small branches of the left renal vein (LRV), sampling of the superior TDV1 branch near the renal tumor yielded the highest enrichment of CTCs of all the vascular beds, followed by the left and right internal iliac veins, both TPV1s draining the bladder containing the second synchronous urothelial tumor (**Figures 6B, C**). CTC levels in the right renal vein (RRV), which did not drain a known solid tumor, were comparable to those in a peripheral sample (PE).

### miRNA in extracellular vesicles (EVs) (Patients 1, 5, and 6)

We next assessed a panel of common cancer-associated microRNAs (listed in Materials and Methods) from each vascular location blood sample, and in four different EV fractions of that sample separated by size using differential ultracentrifugation. The microRNA extraction and quantitative RT-PCR analysis method are described in the Materials and Methods section. The ultracentrifuge size fractions in which specific microRNAs were detected are mentioned in the figure legends.

**Figure 7:**
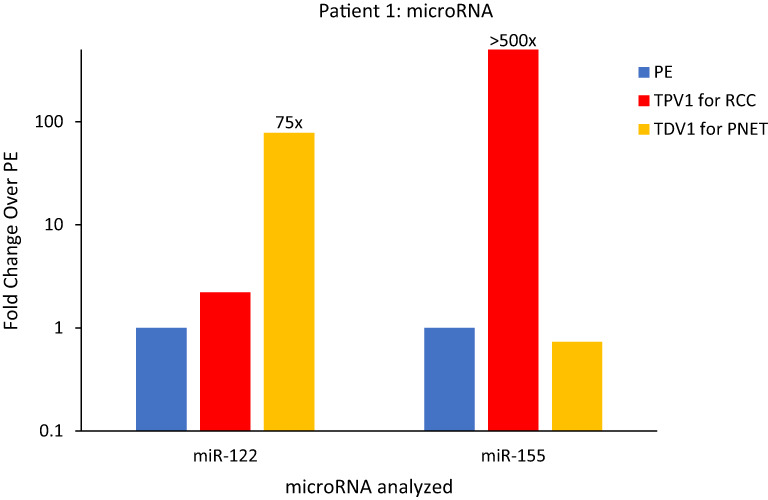
MicroRNA analysis for Patient 1’s PNET and RCC TDVs. miR-122 levels were over 75-fold higher in the TDV1 for the PNET, the portal vein (PV, in yellow) compared to a peripheral sample (PE, in blue) (all 2500P fraction). The TPV1 for the RCC, the right renal vein (RRV, in red) had over 500-fold higher levels of miR-155 over PE (all 110kP fraction). The y-axis is on a log scale.

The TDV1 of the PNET of Patient 1, showed higher concentrations of miR-122 compared to samples from peripheral (>75-fold) and other vascular compartments (**Figure 7**). Notably, miR-122 is associated with indolent tumor biology, similar to Patient 1’s low-grade PNET (31). On the other hand, the TPV1 of the RCC showed higher levels of miR-155 relative to the peripheral sample (>500-fold) (**Figure 7**). While these clearly much higher in Patient 1’s TDV/TPV samples compared to peripheral specimens, the fold changes noted are approximate (without standard error of the mean) due to the unavailability of replicate samples.

We also found miR-155 to be concentrated within Patient 1’s RCC tissue needle biopsy relative to non-tumor renal tissue by 15-fold (**Supplementary Figure 4**). High levels of miR-155 in Patient’s TPV1 and tissue samples is consistent with its known association to renal cell carcinoma (32, 33). None of the microRNAs found elevated in the TDV/TPV of Patient 1 was enriched within blood samples from similar vascular compartments (PV or renal veins) from control patients without known malignancies (**Supplementary Figure 5**). The enrichment of tumor type-related microRNAs in TDV/TPV of Patient 1 was consistent with our findings in certain other patients, as described below.

#### Patient 5

(i) For neck mass, superior vena cava (SVC)=TPV4, and (ii) for abdominal wall mass, infrarenal inferior vena cava (IIVC)=TPV4.

**Figure 8:**
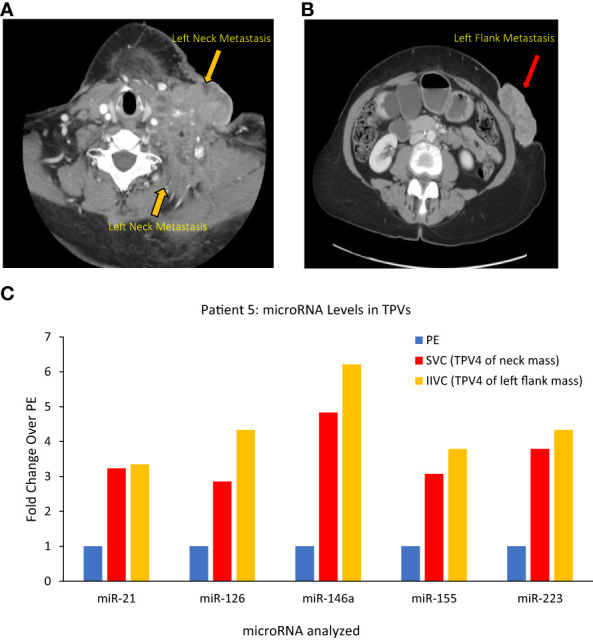
Images of Patient 5’s left neck mass **(A)**, left flank/abdominal wall mass **(B)**, and microRNA analysis of tumor proximal vein (TPV) samples **(C)**. Higher levels of miR-21, miR-126, miR-146a, miR-155 and miR-223 were present in the superior vena cava (SVC, in yellow) and infrarenal inferior vena cava (IIVC, in red) that drain downstream from the neck mass and the left abdominal wall mass, respectively, compared to a peripheral sample from the left hand (PE) (all 2500P fraction). Of note, the SVC and the IIVC are downstream of the neck and abdominal wall masses, respectively, and can be considered TPV4 (not the immediate TDVs) for these masses. In spite of a dilution in tumor-associated miRNA levels expected in these samples, they were still higher than peripheral levels.

Patient 5 was a 70-year-old female who had undergone a total abdominal hysterectomy and bilateral salpingo-oophorectomy after being diagnosed with a high-grade serous papillary ovarian carcinoma stage IIIB (**Table 1**). Two years after the surgery, she presented with metastatic disease recurrence with a left supraclavicular mass (**Figure 8A**) and a left upper abdominal wall mass (**Figure 8B**). In terms of vascular anatomy, the neck and abdominal wall masses would be first drained by their most proximal TDV1s, followed downstream by the left subclavian vein (TPV2), the left brachiocephalic vein (TPV3) and then the superior vena cava (SVC; TPV4). Similarly, the abdominal wall mass would be drained by its most proximal TDV1, which in turn would be followed downstream by the left external iliac vein (TPV2), the left common iliac vein (TPV3) and then the infrarenal inferior vena cava (IIVC; TPV4). We collected samples from the TPV4s for the neck and abdominal wall masses, as well as the left common femoral vein (LCFV) and the left hand (peripheral sample, PE). miRNA analysis as expected revealed that TPV4 samples were substantially enriched for miRNAs 21, 126, 146a, 155 and 223, compared to both the LCFV (not directly in the path of tumor drainage similar to peripheral sample, data not shown) and peripheral blood samples (**Figure 8C**). Several of these microRNAs are associated with ovarian cancer; for e.g., miR-21 is often referred to as a diagnostic biomarker, and miR-146a is considered a marker of sensitivity to chemotherapy (34–38). Notably, the 4^th^ order TPVs that drain downstream of the left neck and abdominal wall masses were likely to be diluted in tumor-associated biomarkers downstream from the TDVs. Yet they showed substantial signal enrichment relative to the peripheral sample. This is in contrast to the rapid dilution in CTC levels in downstream TPVs and may reflect an intrinsic and unique feature of the microRNA/EV signal relative to CTCs.

#### Patient 6

Main branch of the right hepatic vein (RHV)=TPV1 of the metastatic masses in the right hepatic lobe.

**Figure 9:**
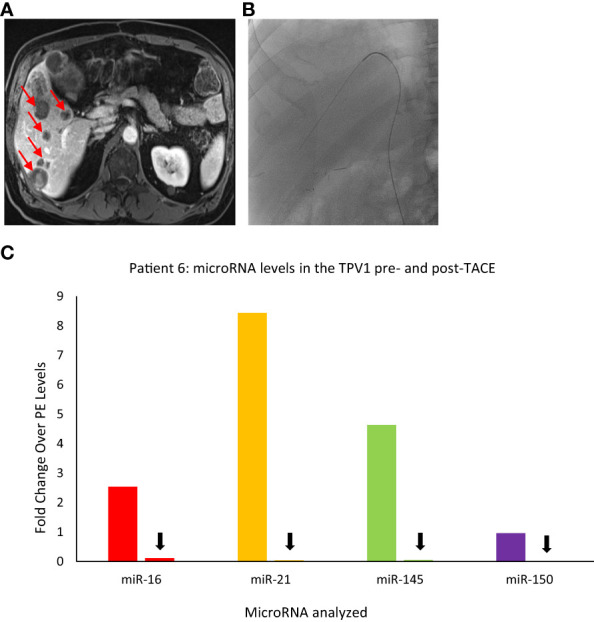
Images of Patient 6’s liver metastasis **(A)**, intra-procedural catheter access **(B)**, and microRNA analysis of TPV samples pre- and post-trans-arterial chemoembolization (TACE) **(C)**. miR-16, miR-21, miR-145, and miR-150 levels were higher in the right hepatic vein (RHV, the TPV1 for the liver mass) before TACE and dropped down after TACE. For this patient, pre- and post-TACE levels in the left common femoral vein (LCFV) were similar for all miRNAs tested (data not shown), and therefore, LCFV was considered equivalent to to a peripheral vein (PE). Data is shown as fold change over pre-TACE PE levels for each miRNA assayed. All samples are from the microsomal RNA 2500P fraction. Red arrows in **(A)** indicate some of the metastatic masses in the liver and black arrows in **(C)** indicate drop in post-TACE levels of microRNAs in the TPV1 samples.

Similar to the substantial immediate reduction in the CTC levels upon selective embolization as noted for Patient 3, TDV/TPV levels of certain microRNAs may also reflect appropriate targeting and reduced tumor arterial perfusion. Patient 6 was a 58-yr-old male with a gastrointestinal stromal tumor (GIST) with hepatic metastases (**Figure 9A**) who underwent a TACE procedure targeting right hepatic lobe *via* the right hepatic artery. Left common femoral vein (LCFV) and TPV1 samples both before and after TACE were analyzed for the levels of various microRNAs in our panel (catheter access of the RHV in **Figure 9B**). The results showed that before TACE, there were higher levels of miR-21 (around 13-fold), miR-145 (around 7-fold), miR-16 (around 4-fold) in the TPV1 compared to LCFV, which was considered the peripheral vein in this case (**Figure 9C**). Immediately after TACE performed the same day, the levels of these microRNAs in the TPV1 dropped to or below peripheral levels, while peripheral levels did not change appreciably post-TACE. This is similar to the substantial and immediate post-TACE drop in CTC levels in the TDV1 of Patient 3, probably because the embolization of the arterial inflow leads to decreased perfusion of the solid hepatic tumor, and thus, less perfusion-related release of either CTCs or EVs/microRNA into the venous outflow immediately post-treatment.

#### EV microscopy

To verify that the ultracentrifuge fractions consisted of the expected sizes of EVs, the same fractions assessed for miRNA levels from Patient 1 were evaluated by transmission electron microscopy (EM) imaging, including the peripheral (PE) and TDV1 samples of the PNET. As anticipated, the 2500P fraction contained larger EVs, including microvesicles and apoptotic bodies (150-1000 nm), often in bigger clusters close to the tumor relative to peripheral samples, in addition to the smaller EVs (exosomes 40-100 nm) (**Supplementary Figure 6**). A similar pattern was noted in the samples from the TDV1 (RRV) samples of the RCC from Patient 1 (data not shown). The 10kP and 110kP fractions, as expected, contained predominantly the smaller exosomes (data not shown). Similarly, we also noted larger vesicles and clusters in the EM images of Patient 8’s tumor-draining ARHV (TPV2; see details regarding Patient 8 in the ctDNA section below) sample that were not seen in a comparable sample from Control Patient 2 taken from the same vascular bed (**Supplementary Figure 7**).

### ctDNA – Mutant allele fraction analysis (Patients 1, 5, 7, and 8)

In general, unlike CTC and microRNA levels, most ctDNA mutations were detected at somewhat similar steady-state levels in blood samples from various vascular beds in our study participants. A few examples are described below: Patient 8 is a typical case while Patients 1, 5, and 7 are exceptions. Variant allele frequency (fraction) (VAF) and the number of mutant allele copies per ml of whole blood analyzed are provided for the specific gene mutations.

In addition to CTC levels and microRNA analysis, we also examined ctDNA from Patient 1’s peripheral and tumor-draining vascular compartments and compared them to known germline and RCC-somatic mutations detected *via* tissue biopsy of the PNET and right renal mass (see Materials and Methods for more details). The PNET tumor tissue revealed no somatic mutations, but the RCC tissue harbored somatic mutations in ERBB2, VHL, and PTEN (**Supplementary Table 1**). Unlike CTCs and miRNA, levels of ctDNA carrying two RCC-specific somatic mutations (proven by tissue biopsy) were not enriched in the samples derived from the right renal vein (RRV) the TPV1 for the RCC (please refer to pre-cryoablation (pre-CA) values in **Supplementary Table 2**).

**Figure 10:**
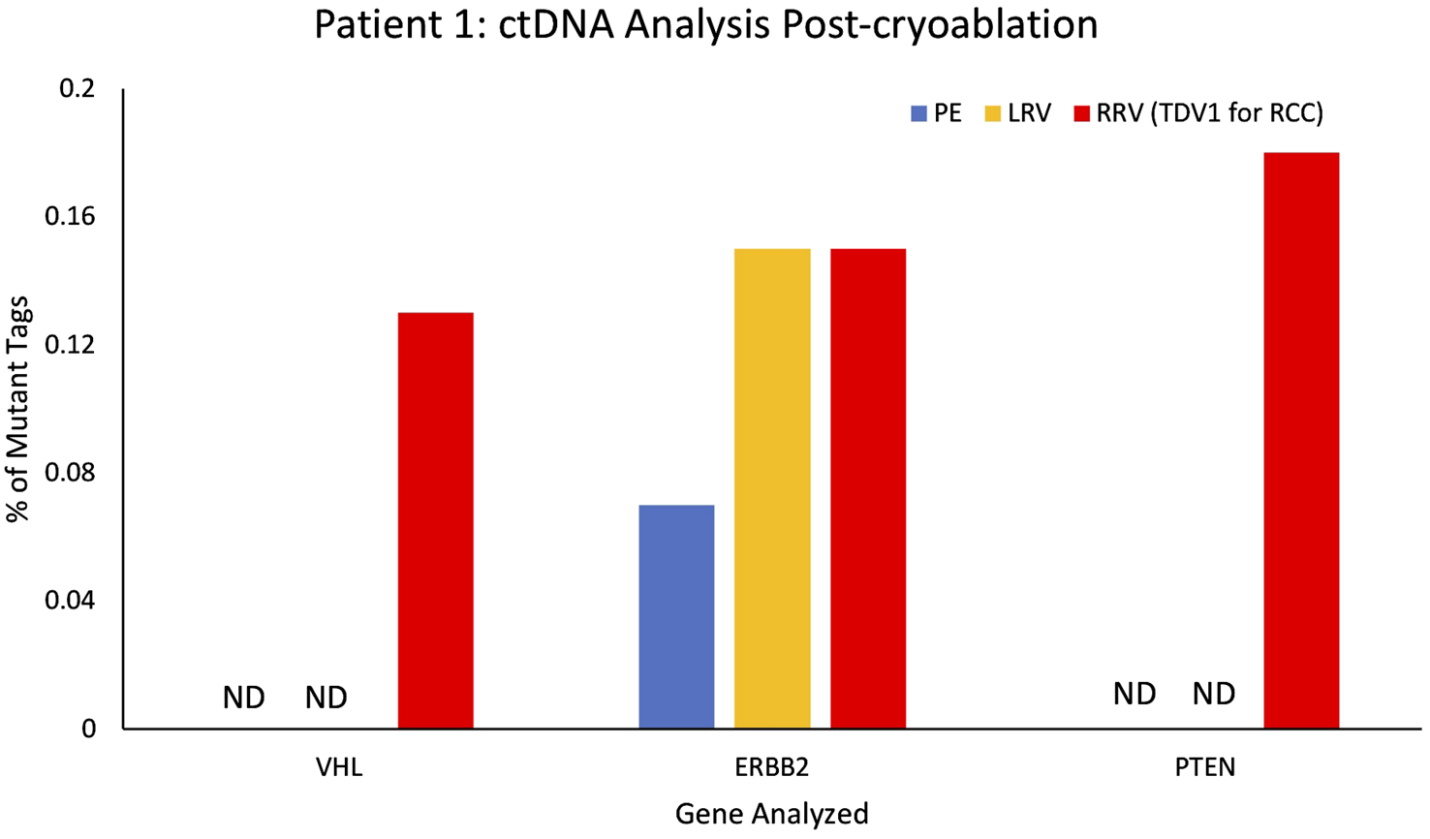
ctDNA analysis for Patient 1 from peripheral and tumor-proximal vein samples, post-cryoablation of a renal cell carcinoma (RCC). Higher variant allele frequencies (VAFs) were seen in the samples from the tumor-proximal right renal vein (RRV, TPV1 for the RCC, in red) compared to peripheral vein (PE, in blue), post-cryoablation, for VHL (87V>P), ERBB2 (424V>L) and PTEN (F215Lfs*6 NS). VAF for ERBB2 (424V>L) was comparable in the RRV and the left renal vein (LRV, in yellow). ND – not detected, VAF – variant allele frequency.

However, this pattern changed after a cryoablation (CA) procedure for Patient 1’s RCC (pre-CA tumor in **Supplementary Figure 8A**). Patient 1 was not a surgical candidate; therefore, the stage IB RCC was successfully cryoablated with curative intent with 6 ablation probes and thorough coverage for margins by intraprocedural imaging. A CT scan performed at a 3-month follow-up visit after CA did not show any enhancing residual tumor within the ablation bed in the right kidney (**Supplementary Figure 8B**). However, a blood sample from the TPV1 for the RCC drawn at the same visit, picked up subtle low-level increases in mutational abundance for known RCC somatic mutations. These included: VHL(p.87V>P) (VAF 0.13%; 0.8 copies/ml), ERBB2(p.424V>L) (VAF 0.15%; 1.2 copies/ml), and PTEN(p.F215Lfs*6) (VAF 0.16%; 1.2 copies/ml) genes (found earlier in the RCC tissue biopsy by whole exome sequencing; **Supplementary Table 1**) that were either undetectable or present in lower levels in the peripheral blood samples of the same date (**Figure 10**; **Supplementary Table 2**). Subsequent CT imaging at a 7-month follow-up visit confirmed the recurrence of the tumor within the ablation bed in the right kidney (**Supplementary Figure 8C**), suggesting that the ctDNA analysis of the TPV1 sample acquired post-CA had provided an early detection of residual tumor in the treated area before it was apparent in the peripheral blood sample same day and eventually picked up by the CT scan 4 months later.

**Figure 11:**
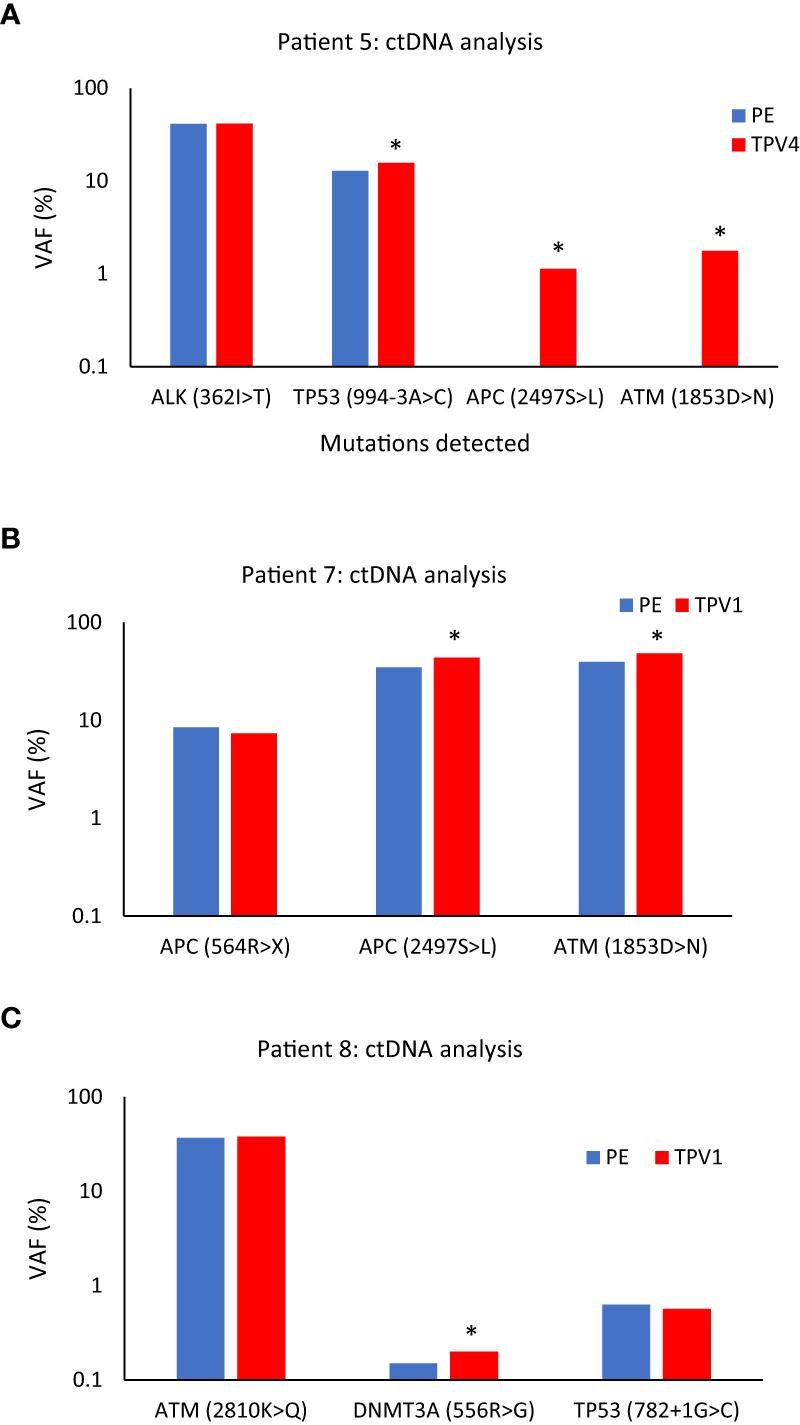
ctDNA mutational analysis for Patients 5 **(A)**, 7 **(B)**, and 8 **(C)** in peripheral versus tumor-draining/proximal veins. **(A)** Higher variant allele frequencies (VAFs) were seen for TP53 (994-3A>C), APC (2497S>L), and ATM (1853D>N) in the superior vena cava (SVC, TPV4) sample of Patient 5’s neck mass compared to peripheral vein (PE) samples. **(B)** VAFs for APC (2497S>L), and ATM (1853D>N) were higher in the ctDNA from the middle hepatic vein 1 (MHV1, TPV1) sample of Patient 7’s segment 8 lesion than a PE sample. **(C)** VAF for DNMT3A (556R>G) was higher in the anterior right hepatic vein (ARHV, TPV1) sample of Patient 8’s segment 6 mass compared to PE. An asterisk (*) indicates increases in VAFs in TDV/TPV samples over PE; it does not imply statistical significance. PE samples are in blue, and the TDV/TPV samples are in red. VAF – variant allele frequency. The y-axis is on a log scale.

As described previously, Patient 5 had a serous ovarian carcinoma that had metastasized to the left neck and left abdominal wall (**Table 1**). In this patient, two mutations: APC(p.2497S>L) with VAF 1.13%, 14.9 copies/ml, and ATM(p.1853D>N) with VAF 1.76%, 10.9 copies/ml, that were not seen in the peripheral vein or other vascular beds, were detected in a sample from TPV4 of the left neck mass. VAF for a mutation in the TP53 gene (p.994-3A>C) was detected at a 21.7% higher level in the TPV4 sample (70.9 copies/ml) compared to a peripheral specimen (**Figure 11A**). Even with the expected dilution in downstream flow from the TDV1 to the point of sampling at TPV4, it was still enriched for these ctDNA mutations compared to the peripheral vein.

#### Patient 7

Middle hepatic vein 1^st^ order branch MHV1=TPV1 for segment 8 lesion, MHV2 is similar to peripheral vein (PE), and right hepatic vein RHV is similar to peripheral vein (PE).

Next is the case of Patient 7, who had advanced sigmoid adenocarcinoma with a partially treated segment 8 liver lesion, in addition to retroperitoneal metastases and lung nodules (**Supplementary Figures 9A, B**). The patient’s hepatic segment 8 lesion would be drained by the middle hepatic vein (MHV) and normal liver parenchyma without tumors drained by the right hepatic vein (RHV). We analyzed samples from the peripheral vein, two potential 1^st^ order branches of the MHV (MHV1 and MHV2), and the right hepatic vein (RHV) sample (intraprocedural catheter access images in **Supplementary Figures 9C–E**) for certain ctDNA mutations. The MHV1 sample, was most likely the TPV1 draining the segment 8 lesion. The ctDNA extracted from this TPV1 sample had a 25.4% higher VAF for the APC(p.2497S>L) mutation (379.6 copies/ml) and a 22.2% higher VAF for the ATM(p.1853D>N) mutation (268.4 copies/ml) than matched peripheral samples (**Figure 11B**).

#### Patient 8

Accessory right hepatic vein (ARHV) anatomical variant=TPV1 branch

One more sample in the ctDNA analysis dataset belonged to Patient 8. Patient 8 had been diagnosed with a poorly differentiated pancreatic adenosquamous carcinoma with larger segment 8 (2.5 cm) and smaller segment 6 (1.5 cm) hepatic metastases (**Supplementary Figure 10A, B**). The vascular beds sampled are listed in **Table 1**. A TPV for the segment 6 metastatic mass was the accessory right hepatic vein (ARHV; **Supplementary Figure 10C, D**), which was a unique anatomical variant that on imaging seemed to be a TPV1 branch draining this small ~ 1.5 cm lesion. ctDNA extracted from this TPV1 sample mostly showed comparable levels of mutations as the matched peripheral sample (**Figure 11C**). However, as described earlier, Patient 8’s TPV1sample showed enrichment of EV clusters compared to a PE sample (**Supplementary Figure 7**).

Taken together, the above results suggest that barring a few exceptions that need to be more rigorously verified, the dynamics of release and/or distribution of ctDNA in the TDVs/TPVs might be different from EVs or CTCs (more in the Discussion section). VAFs and mutant copies/ml whole blood for all the ctDNA results mentioned above for Patients 5, 7, and 8, including these values for the corresponding peripheral samples, are listed in **Supplementary Table 3**.

### Proteomics/biochemical marker analyses (Patients 1 and 9)

**Figure 12:**
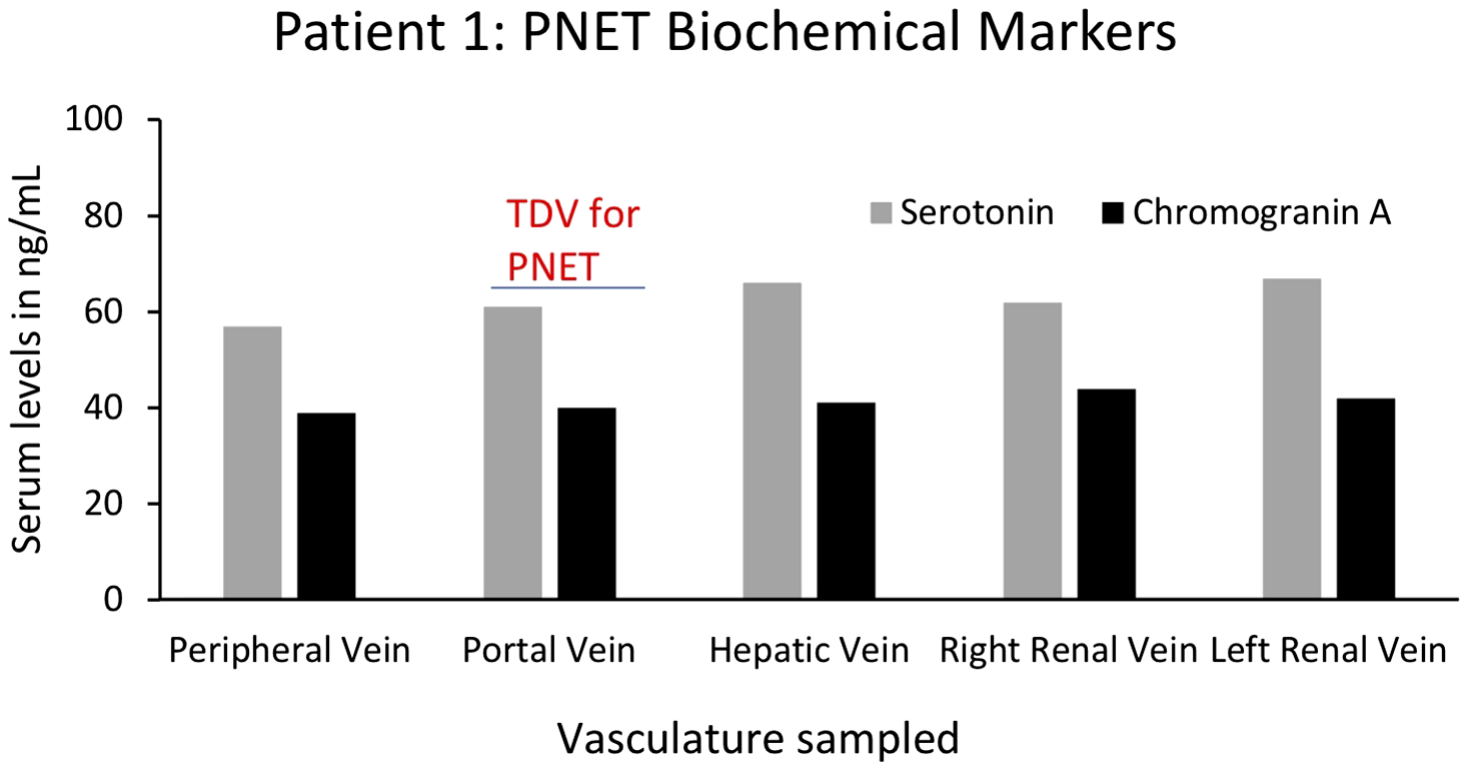
Serum levels of biochemical markers serotonin and chromogranin A for Patient 1’s PNET in tumor-draining and peripheral veins. Levels of known PNET biomarkers, serotonin (grey bars) and chromogranin A (black bars), were not substantially different in blood samples from different vascular venous bed samples and were not enriched in the portal vein, the TDV1 for the PNET. It should be noted that sandostatin (200µg) was administered prior to procedures (that included endovascular venous sampling sessions), to limit serotonin release from the PNET during portal vein stenting procedures and this could have impacted TDV levels of this biomarker.

For yet another oncological biomarker, we looked at proteins/biochemical markers that are known to be associated with particular tumors in two of our patients (1 and 9), to examine if we could detect higher levels in the TDVs compared to the periphery. First, we analyzed the levels of serotonin and chromogranin A in Patient 1’s tumor-draining and peripheral vein samples. Both serotonin and chromogranin A are known tumor markers secreted by pancreatic neuroendocrine tumors (39, 40). Patient 1 was known to have elevated levels of both clinically; therefore, these markers were being tracked by the oncology team as surrogates of the PNET tumor burden. Both serotonin and chromogranin A were detectable at equivalent levels in all vascular compartments in Patient 1, without substantial enrichment within samples collected from the TDV1 of the pancreatic mass, the portal vein (**Figure 12**). Of note, the patient was given 200µg of sandostatin prior to each imaged-guided procedure, which is also when the endovascular blood samples were obtained. Sandostatin was given to minimize risks associated with serotonin release from the PNET during portal vein stenting procedures, and therefore, *active* serotonin and chromogranin A release from the tumor into the TDV1 during sampling is expected to be minimal and could explain the lack of enrichment in the TDV1 during sampling (41).

#### Patient 9

Right hepatic vein (RHV)=TPV1.

For Patient 9, who had a diagnosis of an acinar cell carcinoma of the pancreas with substantial right hepatic lobe (segment 6/7) liver metastasis (**Table 1**), the right hepatic vein (RHV=TPV1) and peripheral blood samples were analyzed for lipase levels before two separate TACE procedures. In these measurements, lipase levels in the TPV1 sample (3516 U/L) were similar to the peripheral vein sample values (3675 U/L) (**Supplementary Figure 11**). However, the peripheral lipase values started dropping gradually from the next day (2752 U/L) and kept declining over the course of the next few weeks to 808 U/L after the second TACE (**Supplementary Figure 11**).

## Discussion

For this proof-of-concept study, we analyzed a case series with nine oncology and two control patients. From these patients, we picked a representative set of data comparing samples from traditional peripheral vein sampling to those acquired from tumor-draining vasculature for their cancer-associated biomarker levels and features. By sampling as close to the 1^st^ order TDVs of known solid tumors as possible, we obtained samples prior to systemic dilution by the circulating blood volume and avoided potential sequestration, degradation, or filtration.

A striking observation from our ctDNA analysis was with respect to the early detection of minimal residual disease (MRD) from the TPV1 sample of Patient 1 following cryoablation, at a timepoint when neither the CT imaging nor the peripheral blood samples showed evidence of disease. Since this result was later confirmed by CT imaging, it suggests that targeted liquid biopsy could potentially provide a minimally invasive method for early MRD detection of a treated solid tumor. Several groups, including some from the biotech industry, are developing methods to monitor patient-specific somatic mutations in the ctDNA extracted from peripheral vein samples as surrogates for MRD and tumor recurrence (42). Our study suggests that TDV/TPV samples would offer improved sensitivity in such approaches compared to matched peripheral samples and would be well-ahead of anatomical cross-sectional imaging modalities such as contrast-enhanced CT in early MRD detection.

Levels of specific exosome-derived miRNAs were also markedly elevated in the TDVs and TPVs in a few of our patients as compared to matched peripheral samples, quite similar to CTCs. This is probably because: a) our method represents enriched sampling from the tumor milieu in the vein just downstream from the solid tumor, and b) our results represent tumor-draining versus peripheral vein levels compared within the same patient and from samples obtained at the same time. Therefore, our results are not subject to inter-patient variability, which is significant because microRNAs are not tumor-specific and can be found in patients without malignancies. To our knowledge, this is the first example of such a robust enrichment of tumor-related microRNA/EV signal within venous drainage of solid malignant lesions. As with Patient 3’s CTCs after TACE, levels of specific microRNAs in Patient 6’s TPV samples decreased after embolization targeting a liver metastatic mass. We also confirmed that the peripheral levels of these microRNAs were less affected immediately post-treatment. Tumor necrosis or apoptosis are unlikely explanations for the rapid decline in CTC and microRNA/EV levels within TPVs in these two patients immediately post-TACE (within minutes). The precipitous drop in signal suggests that CTC and EV release into the TDVs was dependent in part on arterial inflow that was substantially reduced when the TACE embolic cocktail was deposited into the arteries supplying the solid tumors. Importantly, this is in contrast to our findings with ctDNA levels, which were in general more stable and at steady state levels in the different vascular beds and not subject to the rapid kinetics seen with CTCs and EVs. This difference might be due to a passive release of ctDNA from necrotic or apoptotic tumor cells at a slower rate into the vasculature leading to steady state levels, versus a faster, active release process for CTCs as well as EVs/microRNA shed by live cells and their related vasculature [a concept reviewed by Stejskal et al. (43)], but these observations need to be validated in a larger sample set.

Similar to ctDNA, Patient 9’s acinar pancreatic carcinoma-related proteomic biomarker, lipase, did not decrease immediately after embolization within the TPV; instead, it dropped gradually in the peripheral circulation in the days and weeks after each of two separate TACE procedures. This suggests that lipase levels were less reflective of arterial perfusion (immediately reduced by the embolization) and more correlated to decreased hepatic tumor burden (gradual decline from chemotherapy deposited during TACE), which was confirmed by MRI. The release kinetics of various biomarkers appear to be different as highlighted by our targeted liquid biopsy technique and comparisons with matched peripheral samples in the same patient. None of the three biochemical markers/proteins we assayed in this study–chromogranin A, serotonin, and lipase–were enriched in the TDVs. However, others have reported greater TDV levels of certain protein markers–such as PSA for prostate cancer monitoring–compared to peripheral blood (20). The possibility of detecting higher levels of proteomic markers using TDV sampling needs further investigation for a broader range of proteomic markers and in other types of solid tumors.

Taken together, our data show the potential of combining modern techniques in interventional radiology with molecular diagnostics to overcome sensitivity limitations of liquid biopsies. The *surgical* and *endoscopic* literature has shown enrichment of CTCs within the TDVs of patients sampled across lung, colon, and pancreatic tumors; however, these invasive procedures are performed under general anesthesia and are usually limited to a single time point of sampling during surgical resection. Additionally, our data shows that it might not be necessary to cannulate the TDV1 itself; instead, a 2^nd^, 3^rd^, or 4^th^ order TPV may be accessed to obtain a sufficiently enriched signal. This is consistent with another study in breast cancer patients where an implanted central venous access system was used to obtain a more signal-enriched sample than peripheral blood (44).

Damascelli et al., recently reported higher numbers of circulating tumor cells (CTCs), greater ctDNA levels, and enhanced detection of tumor-associated mutations (ctDNA) in some of the samples obtained from TDVs in a study with three patients (15). Similar to our findings, they found certain mutations were more abundant in the TDV samples, while others were detected at comparable levels in the TDV and peripheral samples. Our study of a bigger patient cohort showed robust enrichment of CTCs, certain ctDNA mutations, and specific tumor-related microRNAs in TDVs and TPVs, as well as treatment effects in the levels of some of these biomarkers. Another group has reported transcriptomic analysis of single CTCs that showed different expression profiles depending on the vascular bed location from which they were sampled during surgery (TDV, periphery, or other venous and arterial compartments), including changes in markers for epithelial and mesenchymal phenotypes (22). Data from these other groups and ours taken together suggest sampling from a TDV or TPV may provide an enriched source of biomarkers, which can then be combined with downstream molecular analysis to monitor evolving changes in the tumor.

In addition, several limitations in our study should be acknowledged. It had a small cohort of only nine oncology patients done in a heterogenous setting with different types of tumors, sampling locations, stages of disease, treatment status, etc. We did not have replicate samples in many instances or a large enough patient cohort to statistically evaluate many of the variables being studied. As stated at the outset, this was a proof-of-concept study rather than a clinical trial focused on a large platform or a specific signal (e.g., ctDNA or CTC). Nevertheless, we saw a unified result; higher levels of biomarkers, especially CTCs and EVs/microRNA were found in TDVs/TPVs with kinetics more closely corresponding to tumor arterial inflow compared to ctDNA and proteomic marker levels. In this context, it should be noted that Tamminga et al. found CTCs isolated from the tumor-proximal pulmonary vein (using CellSearch) during lung tumor resection to be genomically different from the primary tumor (45). More studies are needed to better understand the genomics, as well as the distribution and release kinetics of CTCs and other tumor biomarkers, to ascertain their relevance to oncological diagnosis and prognosis.

Within the context of the above limitations, our data shows that targeted liquid biopsy is a safe clinical technique (eliminating risks of hemorrhage, pneumothorax or tumor seeding of core needle tissue biopsy of solid tumors) and fast (~15 minutes), it offers the possibility of minimally invasive access to multiple tumors in an individual patient at numerous times over the course of therapy. This would allow for site-specific biomarker evaluation and monitoring in this era of personalized medicine. To our knowledge, this is the first report of a systematic evaluation for a variety of released biomarkers in tumor-draining veins and other venous compartments across various malignancies, solid tumor locations, staging, including pre- and post-locoregional treatment algorithms.

## Data availability statement

The original contributions presented in the study are included in the article/**Supplementary Material**. Further inquiries can be directed to the corresponding author.

## Ethics statement

The studies involving human participants were reviewed and approved by Institutional Review Board, Johns Hopkins University, Baltimore, MD, USA. The patients/participants provided their written informed consent to participate in this study.

## Author contributions

Concept and design: AT, LD. Clinical/experimental procedures and data acquisition: AT, VW, FT. Data analysis and interpretation: AT, SS, FT, BB, AG. Manuscript writing: AT, SS, AG. All authors contributed to the study and approved the submitted version.

## References

[1] Yáñez-Mó M Siljander P R-M Andreu Z Bedina Zavec A Stampe Ostenfeld M Stoorvogel W. Biological properties of extracellular vesicles and their physiological functions. J Extracell Vesicles (2015) 4(27066). doi: 10.3402/jev.v4.27066 PMC4433489 25979354

[2] Castro-Giner F Gkountela S Donato C Alborelli I Quagliata L Y Ng CK. Cancer diagnosis using a liquid biopsy: Challenges and expectations . Available at: www.mdpi.com/journal/diagnostics. 10.3390/diagnostics8020031 PMC6023445 29747380

[3] Heidrich I Ačkar L Mossahebi Mohammadi P Pantel K. Liquid biopsies: Potential and challenges. Int J Cancer (2021) 148(3):528–45. doi: 10.1002/ijc.33217 32683679

[4] Mathai RA Sri Vidya RV Reddy BS Thomas L Udupa K Kolesar J. Clinical medicine potential utility of liquid biopsy as a diagnostic and prognostic tool for the assessment of solid tumors: Implications in the precision oncology (2019). Available at: www.mdpi.com/journal/jcm. 10.3390/jcm8030373 PMC6463095 30889786

[5] Ignatiadis M Sledge GW Jeffrey SS. Liquid biopsy enters the clinic — implementation issues and future challenges. Nat Rev Clin Oncol (2021) 18(5):297–312. doi: 10.1038/s41571-020-00457-x 33473219

[6] Ilié M Hofman P. Pros: Can tissue biopsy be replaced by liquid biopsy? Transl Lung Cancer Res (2016) 5(4):420–3. doi: 10.21037/tlcr.2016.08.06 PMC5009092 27655109

[7] Mohanty A Mohanty SK Rout S Pani C. Liquid biopsy, the hype vs. hope in molecular and clinical oncology. Semin Oncol (2021) 48(3):259–267. doi: 10.1053/j.seminoncol.2021.06.002 34384614

[8] Alix-Panabières C Pantel K. Liquid biopsy: From discovery to clinical application. Cancer Discovery (2021) 11(4):858–73. doi: 10.1158/2159-8290.CD-20-1311 33811121

[9] Choudhury Y Tan MH Shi JL Tee A Ngeow KC Poh J. Complementing tissue testing with plasma mutation profiling improves therapeutic decision-making for patients with lung cancer. Front Med (2022) 9(February):1–11. doi: 10.3389/fmed.2022.758464 PMC8873935 35223889

[10] Page RD Drusbosky LM Dada H Raymond VM Daniel DB Divers SG. Clinical outcomes for plasma-based comprehensive genomic profiling versus standard-of-Care tissue testing in advanced non–small cell lung cancer. Clin Lung Cancer (2022) 23(1):72–81. doi: 10.1016/j.cllc.2021.10.001 34782240

[11] Kunimasa K Nishino K Sato Y Mori M Ihara S Suzuki H. Fragment size and dynamics of EGFR-mutated tumor-derived DNA provide prognostic information regarding EGFR-TKI efficacy in patients with EGFR- mutated NSCLC. Sci Rep (2022) 12(13544). doi: 10.1038/s41598-022-17848-y PMC9360008 35941190

[12] Wood-Bouwens CM Haslem D Moulton B Almeda AF Lee H Heestand GM. Therapeutic monitoring of circulating DNA mutations in metastatic cancer with personalized digital PCR. J Mol Diagnostics (2020) 22(2):247–61. doi: 10.1016/j.jmoldx.2019.10.008 PMC7031679 31837432

[13] Hattori M Nakanishi H Yoshimura M Iwase M Yoshimura A Adachi Y. Circulating tumor cells detection in tumor draining vein of breast cancer patients. Sci Rep (2019) 9(1):1–10. doi: 10.1038/s41598-019-54839-y 31796846 PMC6890763

[14] Reddy RM Murlidhar V Zhao L Grabauskiene S Zhang Z Ramnath N. Pulmonary venous blood sampling significantly increases the yield of circulating tumor cells in early-stage lung cancer. J Thorac Cardiovasc Surg (2016) 151 (3):852–8. doi: 10.1016/j.jtcvs.2015.09.126 26614417

[15] Damascelli B Tichà V Repetti E Dorji T. Beyond standard practice in liquid biopsy: Selective venous sampling. J Vasc Interv Radiol (2021) 32(5):668–71. doi: 10.1016/j.jvir.2021.02.010 33621662

[16] Catenacci DVT Chapman CG Xu P Koons A Konda VJ Siddiqui UD. Acquisition of portal venous circulating tumor cells from patients with pancreaticobiliary cancers by endoscopic ultrasound. Gastroenterology (2015) 149(7):1794–1803.e4. doi: 10.1053/j.gastro.2015.08.050 26341722 PMC4985007

[17] Crosbie PAJ Shah R Krysiak P Zhou C Morris K Tugwood J. Circulating tumor cells detected in the tumor-draining pulmonary vein are associated with disease recurrence after surgical resection of NSCLC. J Thorac Oncol (2016) 11(10):1793–7. doi: 10.1016/j.jtho.2016.06.017 PMC5063509 27468936

[18] Chudasama D Burnside N Beeson J Karteris E Rice A Anikin V. Perioperative detection of circulating tumour cells in patients with lung cancer. Oncol Lett (2017) 14:1281–6. doi: 10.3892/ol.2017.6366 PMC5529936 28789342

[19] Sun Y-F Guo W Xu Y Shi Y-H Gong Z-J Ji Y. Circulating tumor cells from different vascular sites exhibit spatial heterogeneity in epithelial and mesenchymal composition and distinct clinical significance in hepatocellular carcinoma. Clin Cancer Res (2018) 24(3):547–59. doi: 10.1158/1078-0432.CCR-17-1063 29070526

[20] Farrelly C Lal P Trerotola SO Nadolski GJ Watts MM Gorrian CM. Correlation of peripheral vein tumour marker levels, internal iliac vein tumour marker levels and radical prostatectomy specimens in patients with prostate cancer and borderline high prostate-specific antigen: A pilot study. Cardiovasc Intervent Radiol (2016) 39(5):724–31. doi: 10.1007/s00270-016-1322-5 26957011

[21] Murlidhar V Reddy RM Fouladdel S Zhao L Ishikawa MK Grabauskiene S. Clinical studies poor prognosis indicated by venous circulating tumor cell clusters in early-stage lung cancers. Cancer Res (2017) 77(18):5194–206. doi: 10.1158/0008-5472.CAN-16-2072 PMC5600850 28716896

[22] Sun Y-F Wu L Liu S-P Jiang M-M Hu B Zhou K-Q. Dissecting spatial heterogeneity and the immune-evasion mechanism of CTCs by single-cell RNA-seq in hepatocellular carcinoma. Nat Commun (2021) 12(4091). doi: 10.1038/s41467-021-24386-0 PMC8253833 34215748

[23] Buscail E Chiche L Laurent C Vendrely V Denost Q Denis J. Tumor-proximal liquid biopsy to improve diagnostic and prognostic performances of circulating tumor cells. Mol Oncol (2019) 13(9):1811–26. doi: 10.1002/1878-0261.12534 PMC6717761 31216108

[24] Santasusagna S Moreno I Navarro A Castellano JJ Martinez F Hernández R. Proteomic analysis of liquid biopsy from tumor-draining vein indicates that high expression of exosomal ECM1 is associated with relapse in stage I-III colon cancer. Transl Oncol (2018) 11(3):715–21. doi: 10.1016/j.tranon.2018.03.010 PMC6056757 29660691

[25] Monzo M Martínez-Rodenas F Moreno I Navarro A Santasusagna S Macias I. Differential MIR-21 expression in plasma from mesenteric versus peripheral veins. Med (Baltimore) (2015) 94(1):1–7. doi: 10.1097/MD.0000000000000145 PMC4602834 25569638

[26] Melby JC Spark RF Dale SL Egdahl RH Kahn PC. Diagnosis and localization of aldosterone-producing adenomas by adrenal-vein catheterization. N Engl J Med (1977) 277(20):1050–6. doi: 10.1056/NEJM196711162772002 6059584

[27] Zampetti B Grossrubatscher E Dalino Ciaramella P Boccardi E Loli P. Bilateral inferior petrosal sinus sampling. Endocr Connect (2016) 5:R12–25. doi: 10.1530/EC-16-0029 PMC5002953 27352844

[28] Monticone S Viola A Rossato D Veglio F Reincke M Gomez-Sanchez C. Adrenal vein sampling in primary aldosteronism: Towards a standardised protocol. Lancet Diabetes Endocrinol (2015) 3(4):296–303. doi: 10.1016/S2213-8587(14)70069-5 24831990

[29] Zhao K Patel N Kulkarni K Gross JS Taslakian B. Essentials of insulinoma localization with selective arterial calcium stimulation and hepatic venous sampling. J Clin Med (2020) 9(10):1–14. doi: 10.3390/jcm9103091 PMC7601191 32992761

[30] Kang K Peng X Luo J Gou D. Identification of circulating miRNA biomarkers based on global quantitative real-time PCR profiling. J Anim Sci Biotechnol (2012) 3(1):4. doi: 10.1186/2049-1891-3-4 22958414 PMC3415128

[31] Tsai W-C Wei-Che Hsu P Lai T-C Chau G-Y Lin C-W Chen C-M. MicroRNA-122, a tumor suppressor MicroRNA that regulates intrahepatic metastasis of hepatocellular carcinoma. Hepatology (2009) 49(5):1571–82. doi: 10.1002/hep.22806 19296470

[32] Jung M Mollenkopf H-J Grimm C Wagner I Albrecht M Waller T. MicroRNA profiling of clear cell renal cell cancer identifies a robust signature to define renal malignancy. Mol Oncol (2009) 13(9B):3918–28. doi: 10.1111/j.1582-4934.2009.00705.x PMC4516539 19228262

[33] Shinmei S Sakamoto N Goto K Sentani K Anami K Hayashi T. MicroRNA-155 is a predictive marker for survival in patients with clear cell renal cell carcinoma. Int J Urol. (2013) 20(5):468–77. doi: 10.1111/j.1442-2042.2012.03182.x 23050614

[34] Shi C Zhang Z. Screening of potentially crucial genes and regulatory factors involved in epithelial ovarian cancer using microarray analysi. Oncol Lett (2017) 14(1):725–32. doi: 10.3892/ol.2017.6183 PMC5494615 28693226

[35] Li X Jin Y Mu Z Chen W Jiang S. MicroRNA-146a-5p enhances cisplatin-induced apoptosis in ovarian cancer cells by targeting multiple anti-apoptotic genes. Int J Oncol (2017) 51(1):327–35. doi: 10.3892/ijo.2017.4023 28560455

[36] Paliwal N Vashist M Chauhan M. Evaluation of miR-22 and miR-21 as diagnostic biomarkers in patients with epithelial ovarian cancer. 3 Biotech (2020) 10(3):1–6. doi: 10.1007/s13205-020-2124-7 32206491 PMC7046840

[37] Song KW Zhang QG Tan WB Fang YN. Diagnostic significance of serum miR-26b and miR-21 expressions in ovarian cancer and their associations with clinicopathological characteristics and prognosis of patients. Eur Rev Med Pharmacol Sci (2020) 24(4):1697–703. doi: 10.26355/eurrev_202002_20344 32141536

[38] Hulstaert E Morlion A Levanon K Vandesompele J Mestdagh P. Candidate RNA biomarkers in biofluids for early diagnosis of ovarian cancer: A systematic review. Gynecol Oncol (2021) 160(2):633–42. doi: 10.1016/j.ygyno.2020.11.018 33257015

[39] Prestifilippo A Blanco G Paola M Giuffri D. Chromogranin a and neuroendocrine tumors. Neuroendocr Tumor (2012) Available at: 10.5772/33357.

[40] Sansone A Lauretta R Vottari S Chiefari A Barnabei A Romanelli F. Neuroendocrine gastroenteropancreatic tumors. Cancers (2019) 11 (1113): 1–14 doi: 10.3390/cancers11081113 PMC6721814 31382663

[41] Gupta S. Intra-arterial liver-directed therapies for neuroendocrine hepatic metastases. In: Brown DB, editor. Semin Intervent Radiol (2013) 30 (1):28–38. doi: 10.1055/s-0033-1333651 PMC3700796 24436515

[42] Peng Y Mei W Ma K Zeng C. Circulating tumor DNA and minimal residual disease (MRD) in solid tumors: Current horizons and future perspectives. Front Oncol (2021) 11. doi: 10.3389/fonc.2021.763790 PMC8637327 34868984

[43] Stejskal P Goodarzi H Srovnal J Hajdúch M van ‘t Veer LJ Magbanua MJM. Circulating tumor nucleic acids: biology, release mechanisms, and clinical relevance. Mol Cancer (2023) 22(1):1–21. doi: 10.1186/s12943-022-01710-w 36681803 PMC9862574

[44] Peeters DJE Van Den Eynden GG Van Dam PJ Prové A Benoy IH Van Dam PA. Circulating tumour cells in the central and the peripheral venous compartment in patients with metastatic breast cancer. Br J Cancer (2011) 104(9):1472–7. doi: 10.1038/bjc.2011.122 PMC3101936 21468046

[45] Tamminga M de Wit S van de Wauwer C van den Bos H Swennenhuis JF Klinkenberg TJ. Analysis of released circulating tumor cells during surgery for non-small cell lung cancer. Clin Cancer Res (2020) 26(7):1656–66. doi: 10.1158/1078-0432.CCR-19-2541 31772122

