# Supplementary material for: Endovascular image-guided sampling of tumor-draining veins provides an enriched source of oncological biomarkers
Source: Front Oncol. 2023 Mar 17;13:916196. doi: 10.3389/fonc.2023.916196 (PMC10064007; doi:10.3389/fonc.2023.916196)
Supplement: Supplementary file 1 [file DataSheet_1.docx]

**Supplementary Figures**

A B


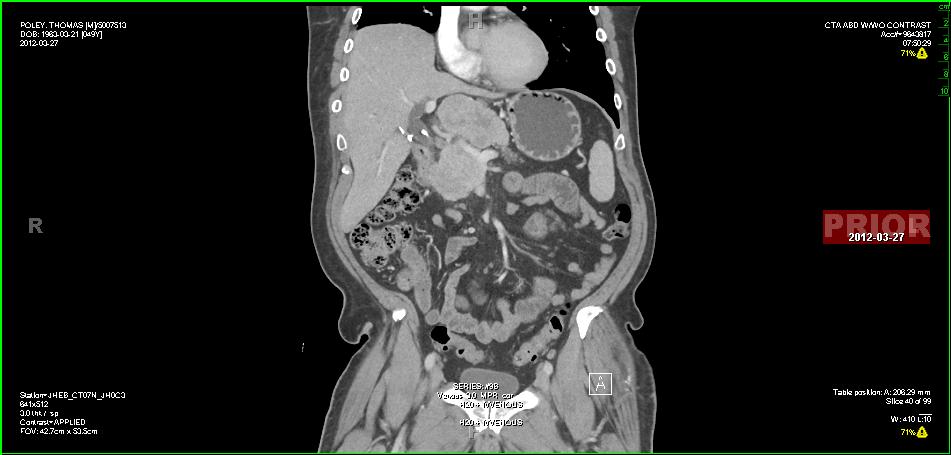

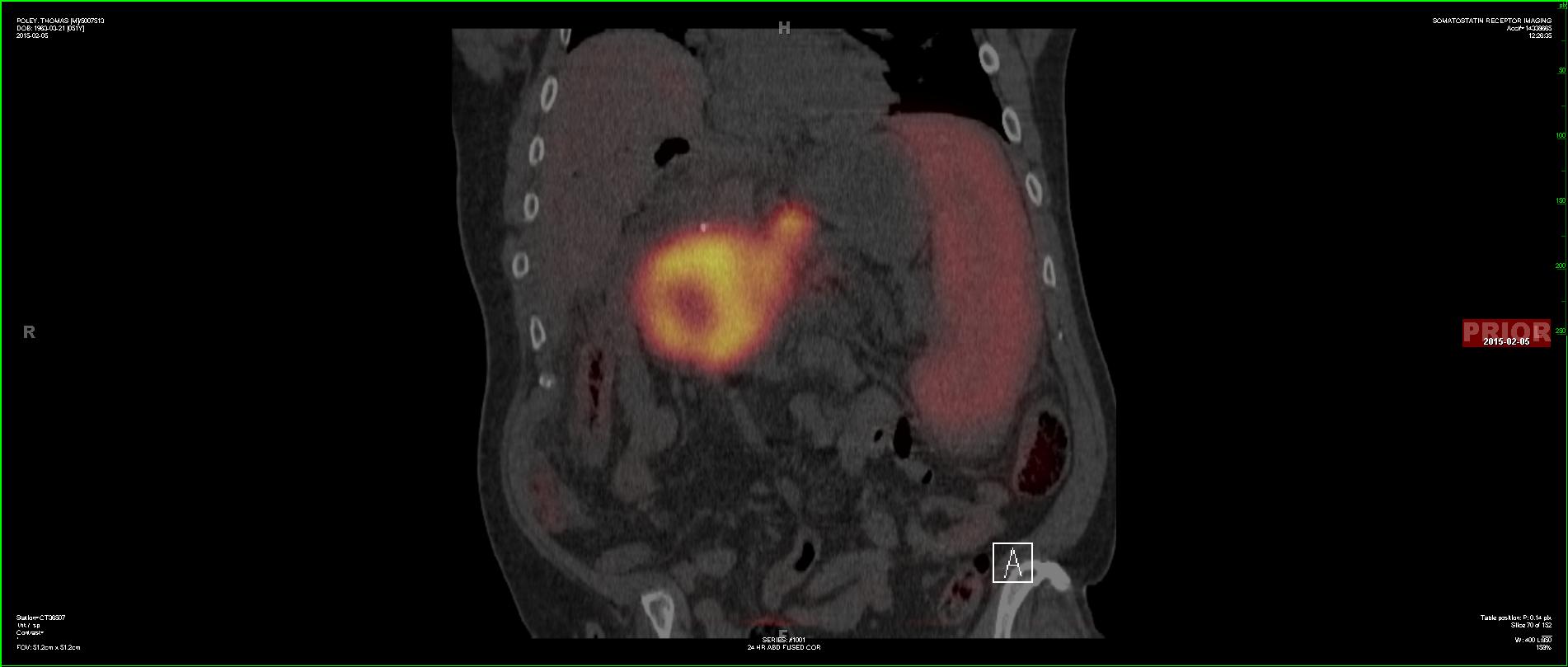


C D


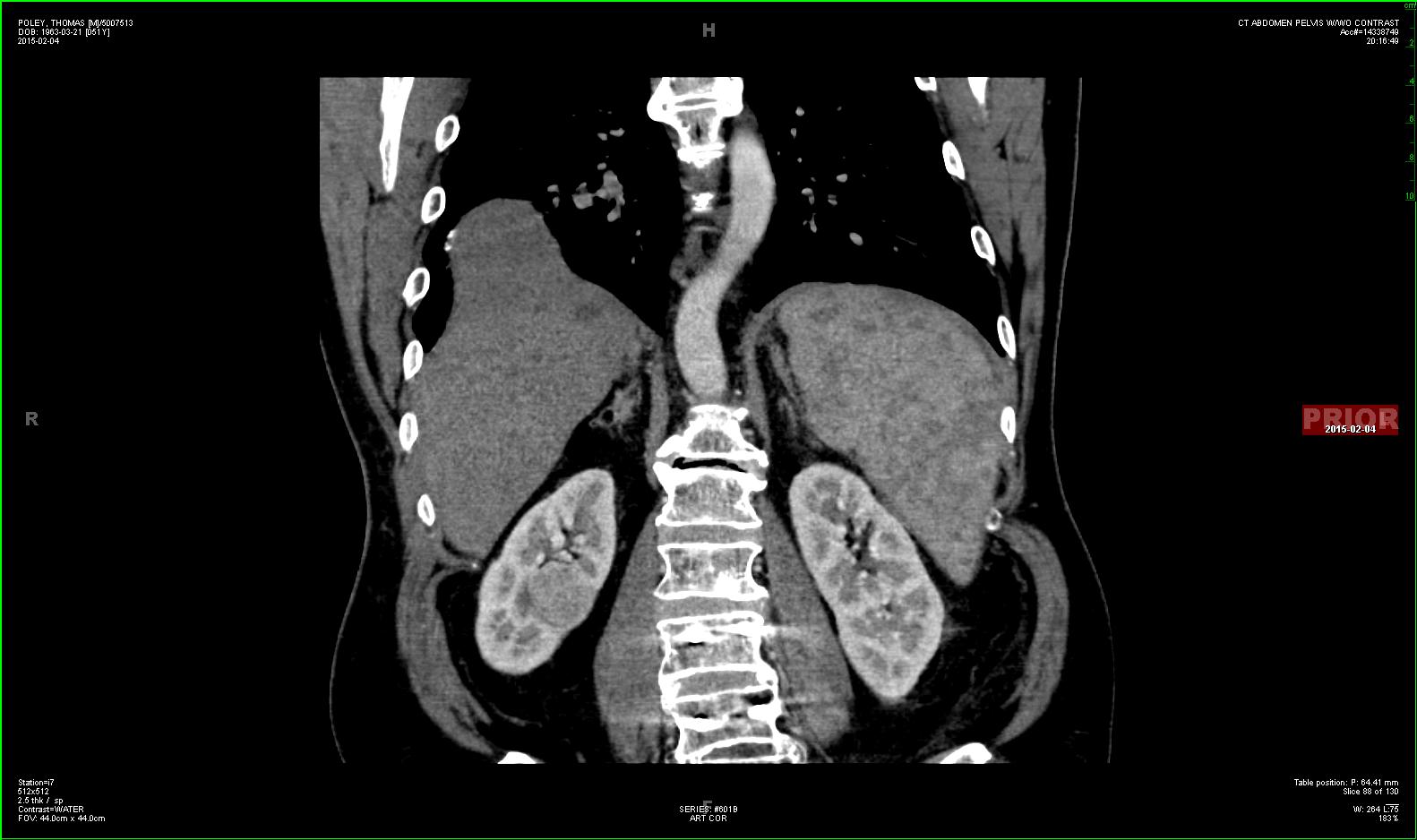


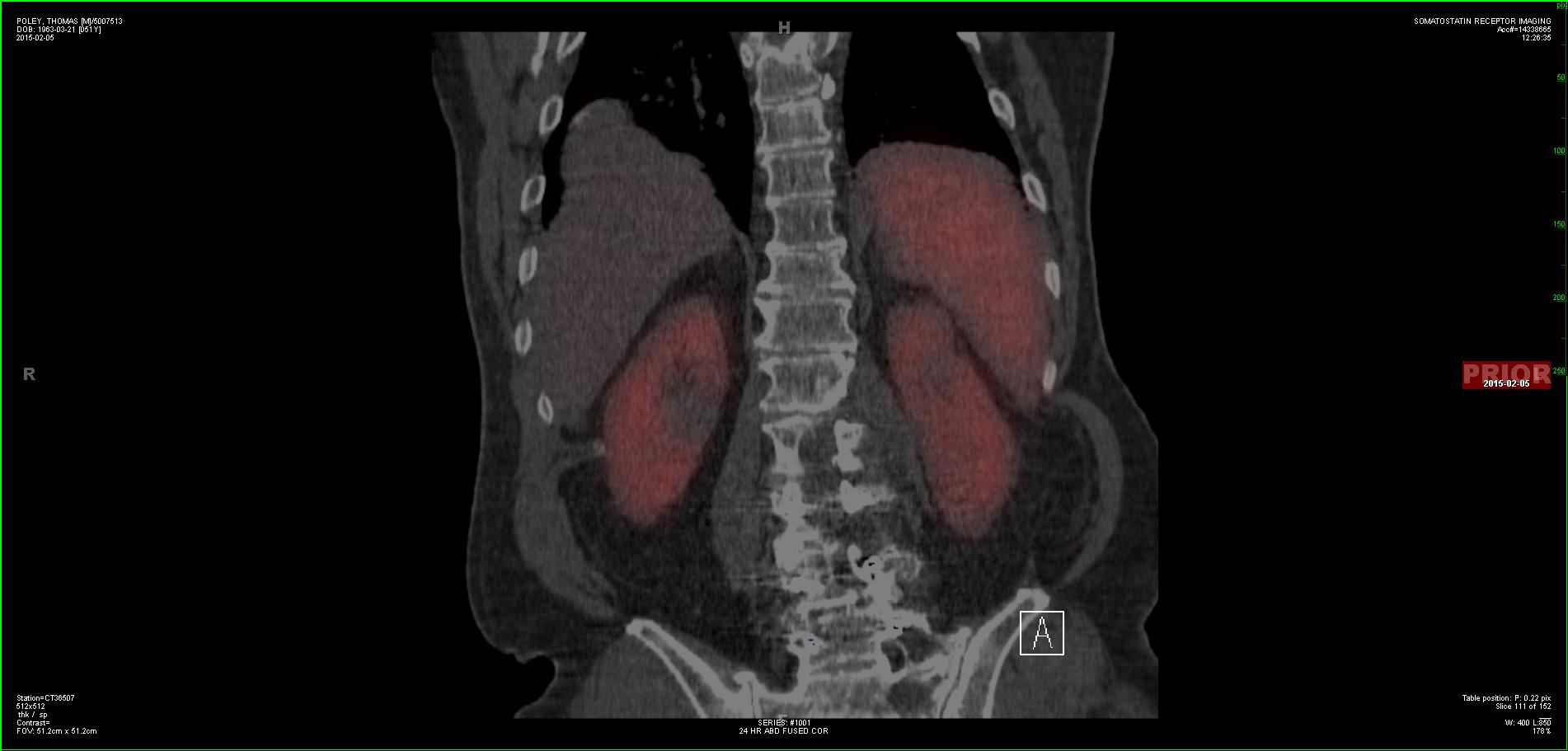


**Supplementary Figure 1:** **Images of the metachronous pancreatic neuroendocrine tumors (PNET) and right renal cell carcinoma (RCC) of Patient 1.** This patient had a large pancreatic mass (**A**) with Indium-111 Octreotide uptake (**B**) that was found to be a well-differentiated PNET. Three years later, an enlarging hypervascular mass was found in the right kidney (**C**) without Indium-111 Octreotide scan tracer uptake (**D**) suggestive of a metachronous renal tumor, confirmed to be an RCC by tissue biopsy. Red circles highlight position of the PNET, and green circles highlight position of the RCC.

A B

C D


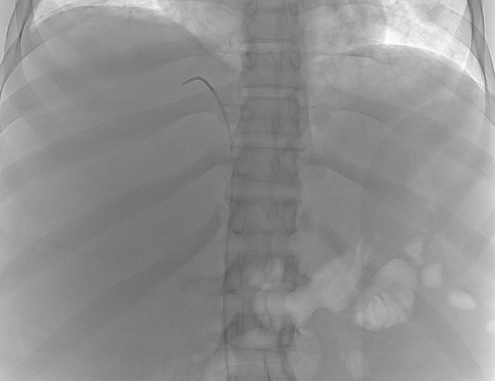

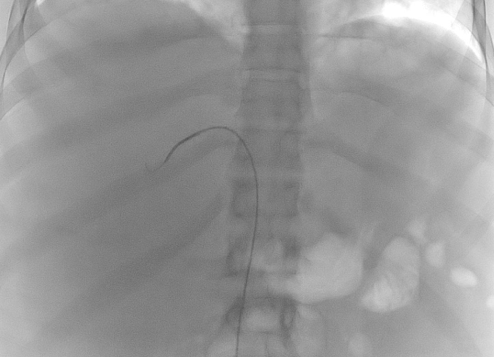


**Supplementary Figure 2:** **Tumor locations and vascular venous compartments for Patient 3.** Images showing the location of the tumor and the main branch of the middle hepatic vein (MHV; TPV1 of segment 5/8 mass) (**A**) and the main (inferior) branch of the accessory right hepatic vein (ARHV; TPV1 of segment 6/7 mass) (**B**) in the patient. Intra-procedural images showing venous sampling of MHV and the ARHV are in (**C)** and (**D)**, respectively. The red arrows indicate the location of the MHV and the ARHV within the axial CT images.

A


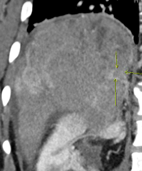

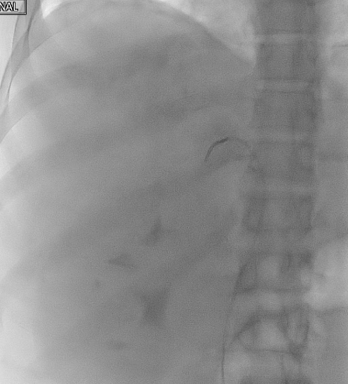


B C


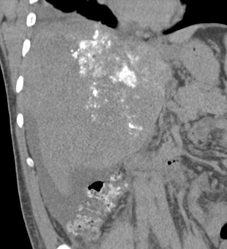


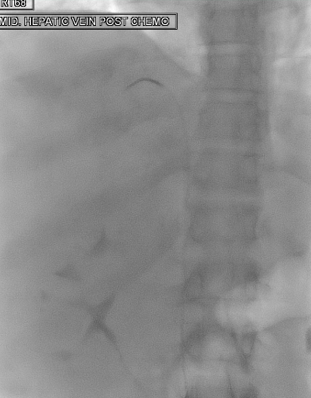


**Supplementary Figure 3:** **Tumor-draining vein (TDV1) sampling of Patient 3 and deposition of chemoembolization cocktail.** (**A)** shows intra-procedural images of the sampling of a small branch of the accessory right hepatic vein (ARHV) which is presumed to be the TDV1 of Patient 3’s segment 5/8 mass. Yellow arrows indicate the TDV1. (**B**) and (**C**) show deposition of the chemotherapy cocktail in the segment 5/8 tumor post-TACE. The red arrows indicate the area of the tumor targeted by the TACE.

**Supplementary Figure 4: miR-155 levels in normal versus renal carcinoma (RCC) tissue of Patient 1.** Tumor tissue had around 15-fold higher levels of miR-155 compared to normal tissue**.** Average of two biopsies is shown**.**

**Supplementary Figure 5: MicroRNA analysis of targeted liquid biopsy blood samples of two control patients without cancer.** A panel of cancer-associated microRNAs and the endogenous control, miR16, did not show substantial fold changes over peripheral levels, within various vascular venous bed samples in two control patients, Control Patient 1 (top panel) and Control Patient 2 (bottom panel). PV, portal vein; LRV, left renal vein; RRV, right renal vein, HV, hepatic vein; and IJV, internal jugular vein.


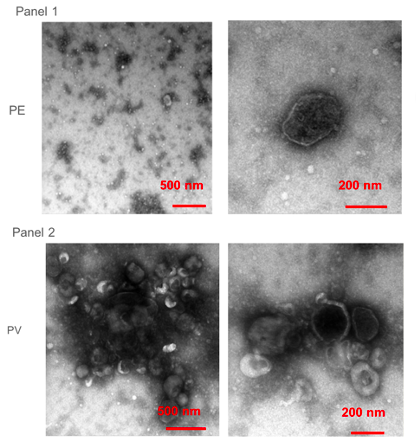


**Supplementary Figure 6: Electron microscopy (EM) images of the 2500K fractions in the tumor-draining portal vein (PV) and peripheral vein (PE) samples of Patient 1.** In general, large extracellular vesicles (EVs) and apoptotic bodies (particulates) with more clusters are found within TDV1 of the tumor (PNET), the portal vein (PV, Panel 2), than in the peripheral (PE, Panel 1) blood samples. Two representative images from each vascular venous bed sample are shown.

A B C


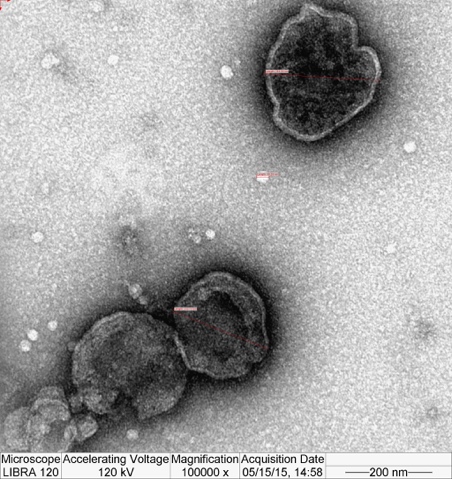

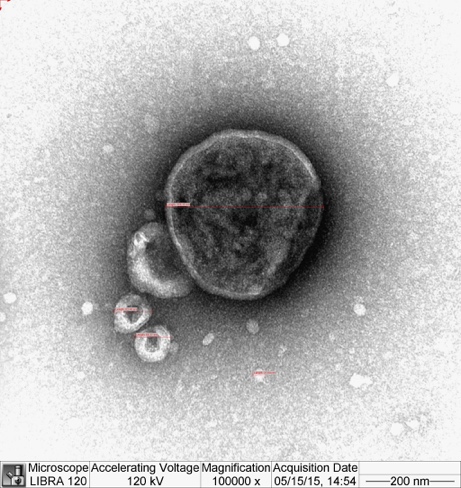

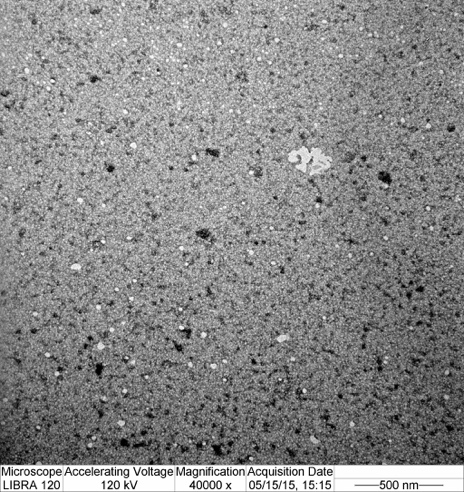


**Supplementary Figure 7: Electron microscopy (EM) images of the 2500K fractions in the accessory right hepatic vein (ARHV) samples of Patient 8 (A) and (B) and Control Patient 2 (C).** ARHV is the tumor-proximal vein (TPV1) for the metastatic liver mass in Patient 8. Large extracellular vesicles (EVs) and apoptotic bodies (particulates) with more clusters are found within ARHV for Patient 8 than in Control Patient 2. (**A**) and (**B**) are at 100,000X and panel C is at 40,000X magnification.

A B C


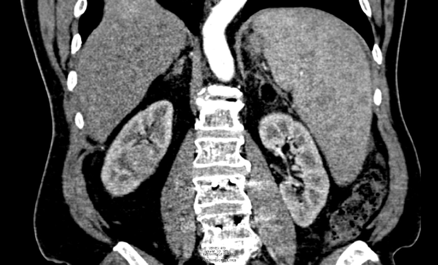


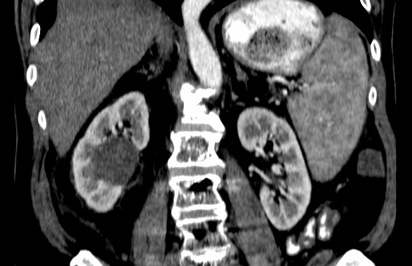

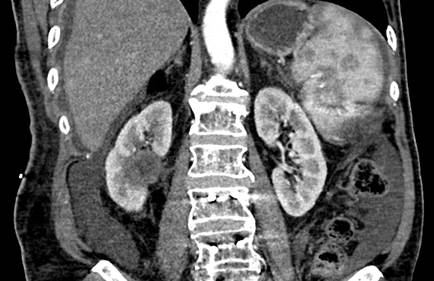


**Supplemental Figure 8: CT images of residual disease in the right kidney of Patient 1 following cryoablation (CA) of the right renal carcinoma (RCC) at the 3-month and 7-month follow-up visits.** The pre-CA scan (**A**) shows enhancing solid mass in right kidney biopsy proven to be RCC. The 3-month scan (**B**) did not detect residual enhancing tumor tissue but ctDNA of a blood sample collected from the right renal vein (TPV1) found elevated levels of ERBB2 and VHL mutations compared to peripheral levels (Figure 12). Recurrence of the disease was confirmed by the CT scan with enhancing soft tissue mass at the ablation bed at the 7-month follow-up (**C**). Yellow arrows indicate the area of the RCC targeted by the CA procedure.

A B


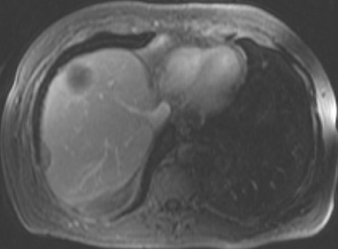


**MHV1**


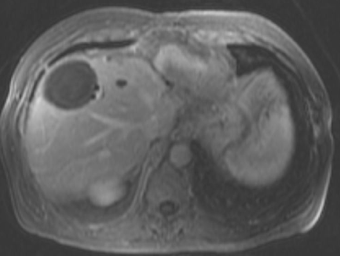


**MHV2**

**RHV**

C D E


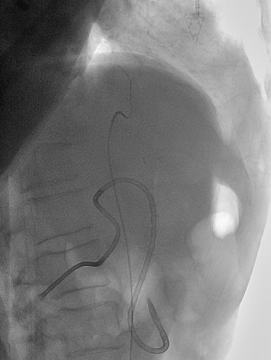

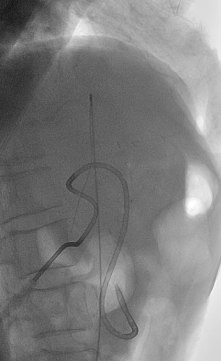

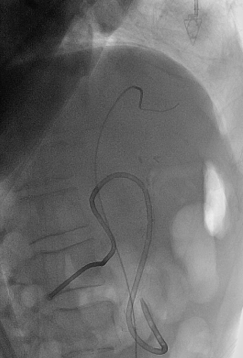


MHV1 RHV MHV2

**Supplementary Figure 9:** **Images of Patient 6’s tumor and tumor-proximal vein sampling.** Patient 6 had sigmoid adenocarcinoma, stage IV, with liver and retroperitoneal metastases and lung nodules. Middle hepatic veins 1 and 2 (MHV1, MHV2) and the right hepatic vein (RHV; TPV1) in the proximity of the liver mass are in **(A)** and **(B)**. Images of intraprocedural catheter access for MHV1, MHV2 and RHV in (**C**), (**D**) and (**E**), respectively.

A B C D

**Supplementary Figure 10: Images of Patient 8’s segment 6 smaller liver metastasis (A, B), tumor-draining vein location (C) and intra-procedural catheter access (D)**. Patient 8’s segment 6 enhancing liver metastasis (~1.5 cm) (red arrows in **A** and **B**) was drained specifically by a variant anatomy accessory right hepatic vein (ARHV, TPV1) (yellow arrow in **C**) that was sampled with a soft endovascular catheter (yellow arrow in **D**).

TPV1 sample and 1^st^ TACE

**Supplementary Figure 11: Serum lipase levels in Patient 9 before and after two trans-arterial chemoembolization (TACE) procedures.** Pre-TACE lipase levels were comparable in the right hepatic vein (RHV; TPV1) (red circle) and peripheral samples (blue circles). Peripheral vein samples had lower lipase levels in the days and weeks following the TACE procedures (arrows indicate dates of these procedures).
