# Supplementary material for: Endovascular image-guided sampling of tumor-draining veins provides an enriched source of oncological biomarkers
Source: Front Oncol. 2023 Mar 17;13:916196. doi: 10.3389/fonc.2023.916196 (PMC10064007; doi:10.3389/fonc.2023.916196)
Supplement: Supplementary file 2 [file DataSheet_2.docx]

| Gene | Gene Symbol | Amino Acid | Mutation Type | Consequence | % VAF |
| --- | --- | --- | --- | --- | --- |
| V-ERB-B2 Avian Erythroblastic Leukemia Viral Oncogene Homolog 2 | ERBB2 | 424 V>L | Substitution | Nonsynonymous Coding | 16 |
| Von Hippel-Lindau Tumor Suppressor, E3 Ubiquitin Protein Ligase | VHL | 87 V>P | Substitution | Nonsynonymous Coding | 26 |
| Phosphate and Tensin Homolog | PTEN | F215Lfs*6 | Deletion | Frameshift | 17 |

**Supplementary Table I: CancerSelect-R^TM^ 88 (Personal Genome Diagnostics) DNA genomic alteration panel results of the right renal mass tissue biopsy.** Six core tissue biopsy samples of the mass were taken with an 18 Gauge Temno (Carefusion) device with a total DNA recovery of 45 ng. The pathologic tumor purity was 70%, and mutational-based tumor purity was 37%. The biologically and clinically relevant mutations found are listed. Normal source of DNA to identify and subtract germline mutations was the patient’s saliva. VAF ­— variant allele frequency.

| Gene | PE % VAF; mutant copies/ml WB | | RRV % VAF; mutant copies/ml WB | | LRV % VAF; mutant copies/ml WB | |
| --- | --- | --- | --- | --- | --- | --- |
|  | Pre-CA | Post-CA | Pre-CA | Post-CA | Pre-CA | Post-CA |
| VHL | 0.29; 2.4 | ND | 0.05; 0.4 | **0.13; 0.8** | 0.1; 1.2 | ND |
| ERBB2 | 0.06; 0.8 | 0.07; 0.8 | 0.04; 8 | **0.15; 1.2** | 0.04; 0.8 | **0.15; 1.2** |
| PTEN | NT | ND | NT | **0.18; 1.2** | NT | ND |

**Supplementary Table II: ctDNA analysis for Patient 1 from peripheral and tumor-draining vein samples, pre- and post-cryoablation (CA) of a renal cell carcinoma.** There was no elevation of ctDNA variant allele frequency (VAF) for VHL(p.87V>P) or ERBB2(p.424V>L) in either the right or the left renal veins (RRV and LRV) compared to a peripheral sample (PE), pre-CA. PTEN(p.F215Lfs*6) VAF was only assayed post-CA due to sample limitations. A higher percentage of all three mutations was seen in the RRV and only ERBB2(p.424V>L) in the LRV, compared to PE, post-CA. Higher values in the TPV1 post-CA compared to pre-CA are in bold. ND – not detected; NT - not tested; WB – whole blood.

| Patient | Gene | Gene Symbol | Mutation Type | Amino Acid (Protein) | Consequence | %VAF; mutant copies per ml in PE | %VAF; mutant copies per ml in TDV/TPV | Change in VAF of TDV/TPV relative to PE |
| --- | --- | --- | --- | --- | --- | --- | --- | --- |
| 5 | Anaplastic lymphoma receptor tyrosine kinase | ALK | Substitution | 362I>T | Non-synonymous coding | 41.3; 740.3 | 41.65; 496.9 | not elevated |
|  | Tumor protein p53 | TP53 | Substitution | 994-3A>C | Splice site acceptor | 12.86; 51.1 | **15.65; 70.9** | **+21.70%** |
|  | Adenomatous polyposis coli | APC | Substitution | 2497S>L | Non-synonymous coding | ND | **1.13; 14.9** | **Only detected in TDV/TPV** |
|  | Ataxia telangiectasia mutated | ATM | Substitution | 1853D>N | Non-synonymous coding | ND | **1.76; 10.9** | **Only detected in TDV/TPV** |
| 6 | Adenomatous polyposis coli | APC | Substitution | 564R>X | Nonsense | 8.44; 43.6 | 7.35; 39.2 | -12.91% |
|  | Adenomatous polyposis coli | APC | Substitution | 2497S>L | Non-synonymous coding | 34.75; 304.8 | **43.57; 379.6** | **+25.38%** |
|  | Ataxia telangiectasia mutated | ATM | Substitution | 1853D>N | Non-synonymous coding | 39.63; 214.8 | **48.41; 268.4** | **+22.15%** |
| 8 | Ataxia telangiectasia mutated | ATM | Substitution | 2810K>Q | Non-synonymous coding | 39.96; 158.8 | 37.93; 196.8 | unclear |
|  | DNA (cytosine-5-)-methyltransferase 3 alpha | DNMT3A | Substitution | 556R>G | Non-synonymous coding | 0.15; 3.2 | 0.20; 3.2 | no change detected |
|  | Tumor protein p53 | TP53 | Substitution | 782+1G>C | Splice site donor | 0.63; 18.4 | 0.57; 12.8 | -9.52% |

**Supplementary Table III: ctDNA mutational analysis for Patients 5, 6, and 8 in peripheral versus tumor-draining/proximal veins.** Higher percentages of variant allele frequency (VAF) were seen for: TP53 (994-3A>C) in the superior vena cava (SVC) sample of Patient 5, APC(p.2497S>L) and ATM(p.1853D>N) in the middle hepatic vein 1 (MHV1) sample of Patient 6. ctDNA mutation levels were not higher in the anterior right hepatic vein (ARHV) sample of Patient 8 compared to a peripheral sample. SVC, MHV1, and ARHV are presumed to be TDV1/TPV1 for Patients 5, 6, and 8’s tumors, respectively (please refer to Table I). TDV/TPV values higher than peripheral values are in bold. ND — not detected.
